# DNA damage triggers tubular endoplasmic reticulum extension to promote apoptosis by facilitating ER-mitochondria signaling

**DOI:** 10.1038/s41422-018-0065-z

**Published:** 2018-07-20

**Authors:** Pengli Zheng, Qingzhou Chen, Xiaoyu Tian, Nannan Qian, Peiyuan Chai, Bing Liu, Junjie Hu, Craig Blackstone, Desheng Zhu, Junlin Teng, Jianguo Chen

**Affiliations:** 1 Key Laboratory of Cell Proliferation and Differentiation of the Ministry of Education, State Key Laboratory of Membrane Biology, College of Life Sciences, Peking University, Beijing, 100871 China; 2 Cell Biology Section, Neurogenetics Branch, National Institute of Neurological Disorders and Stroke, National Institutes of Health, Bethesda, Maryland 20892 USA; 3 College of Life Sciences, Jiangsu Normal University, Xuzhou, 221116 China; 4 Center for Quantitative Biology, Peking University, Beijing, 100871 China; 5 National Laboratory of Macromolecules, Institute of Biophysics, Chinese Academy of Science, Beijing, 100871 China; 6 Department of Genetics and Cell Biology, College of Life Sciences, Nankai University and Tianjin Key Laboratory of Protein Sciences, Tianjin, 300071 China; 7 Laboratory Animal Research Center, Peking University, Beijing, 100871 China

## Abstract

The endoplasmic reticulum (ER) is composed of the nuclear envelope, perinuclear sheets and a peripheral tubular network. The peripheral ER and mitochondria form tight contacts at specific subdomains, which coordinate the functions of the two organelles and are required for multiple cellular processes such as Ca^2+^ transfer and apoptosis. However, it is largely unknown how ER morphology and ER-mitochondria signaling are dynamically regulated under different physiological or pathological conditions such as DNA damage. Here we show that the peripheral, tubular ER undergoes significant extension in response to DNA damage, and that this process is dependent on p53-mediated transcriptional activation of the ER-shaping proteins REEP1, REEP2 and EI24 (alias PIG8). This promotes the formation of ER-mitochondria contacts through EI24 and the mitochondrial outer membrane protein VDAC2, facilitates Ca^2+^ transfer from ER to mitochondria and promotes DNA damage-induced apoptosis. Thus, we identify a unique DNA damage response pathway involving alterations in ER morphology, ER-mitochondria signaling, and apoptosis.

## Introduction

The endoplasmic reticulum (ER) is the largest membranous organelle and performs essential roles in protein synthesis and secretion, Ca^2+^ homeostasis, and lipid metabolism. Morphologically, the ER consists of the nuclear envelope, high density sheets in the perinuclear region, and a peripheral tubular network.^1,2^ Dysregulation of proper ER morphology is associated with various human diseases such as hereditary spastic paraplegia (HSP),^3^ Alzheimer’s disease^4^ and cancer.^5^ Several proteins have been identified to regulate ER morphology. Climp63, kinectin and p180 are important for the formation of ER sheets,^6^ whereas Reticulons (Rtns),^7^ receptor expression enhancing proteins (REEPs),^8^ Atlastins,^9^ and Lunapark (Lnp1)^10^ generate the tubular ER. Tubular ER-shaping proteins of the reticulon and REEP families contain one or more intramembrane hairpin regions consisting of two closely-spanned short transmembrane domains that are proposed to form wedge-like structures within the outer leaflet of the lipid bilayer, stabilizing the high membrane curvature of the ER tubules.^11^ REEP1 and REEP2 (REEP1/2) are both reported to be HSP-related proteins.^8,12^ REEP1 also plays important roles in lipid droplet formation,^13^ ER stress response,^14^ and ER-mitochondria contacts.^15^

The ER and mitochondria are often tightly associated at specific subdomains via tethering mediated by mitochondria-associated ER membrane (MAM) proteins. These contacts enable Ca^2+^ transfer with high efficiency from the ER to mitochondria, which is necessary for mitochondrial metabolism.^16^ However, dramatically increased ER-mitochondria Ca^2+^ flux triggers apoptosis by activating the mitochondrial permeability transition pore and subsequently releasing cytochrome c.^16^ Therefore, ER-mitochondria contacts are critical for determining cell fate. A complex formed by voltage-dependent anion channel 1 (VDAC1), glucose-regulated protein 75 (GRP75), and the inositol-1,4,5-trisphosphate receptor (IP3R), known as the MAM complex,^17^ has been reported to be involved in the response to several stress conditions, such as ER stress and oxidative stress.^17–19^

Upon DNA damage, cells initiate several response pathways that include DNA-PK and ATM/ATR to activate DNA repair, cell cycle arrest and/or apoptosis.^20^ An improper or insufficient DNA damage response can lead to genetic mutations and cancer development.^21^ One key player in the DNA damage response is the tumor suppressor p53, which promotes cell cycle arrest and DNA repair in response to moderate DNA damage, but apoptosis to  severe DNA damage.^22^ Among the p53 target proteins, etoposide-induced protein 2.4 (EI24) (alias p53-induced gene 8 protein, PIG8) is an ER-localized transmembrane protein that was originally reported to be a tumor suppressor^23^ and is frequently lost or mutated in various cancers.^24–27^ EI24 has been reported to inhibit cell growth and promote apoptosis.^28^ Loss of EI24 leads to resistance to DNA damage-induced cell death^29^ and is associated with breast tumor invasiveness.^30^ In addition, in p53-deficient cells, EI24 acts as an E2F target that contributes to cell survival after UV irradiation.^31^ A recent report showed that EI24 associates with the nuclear import machinery and inhibits the nuclear translocation of p53.^32^ Thus, the exact role of EI24 in apoptosis seems complex.

Although the DNA damage response has been studied intensively over the past few decades, the mechanisms whereby DNA damage affects the structures and function of cytoplasmic organelles have only begun to be elucidated.^33^ As ER function is intimately related to the DNA damage response and pathways downstream of p53,^34–36^ we investigated whether the morphology of ER responds to DNA damage. We show that DNA damage triggers tubular ER extension via the p53-mediated expression of REEP1/2 and EI24, and that this facilitates contacts between ER and mitochondria. We further show that under DNA damage conditions, EI24 and VDAC2 mediate the formation of a unique ER-mitochondria contact that promotes mitochondrial Ca^2+^ uptake and apoptosis.

## Results

### DNA damage induces tubular ER extension

**Fig. 1:**
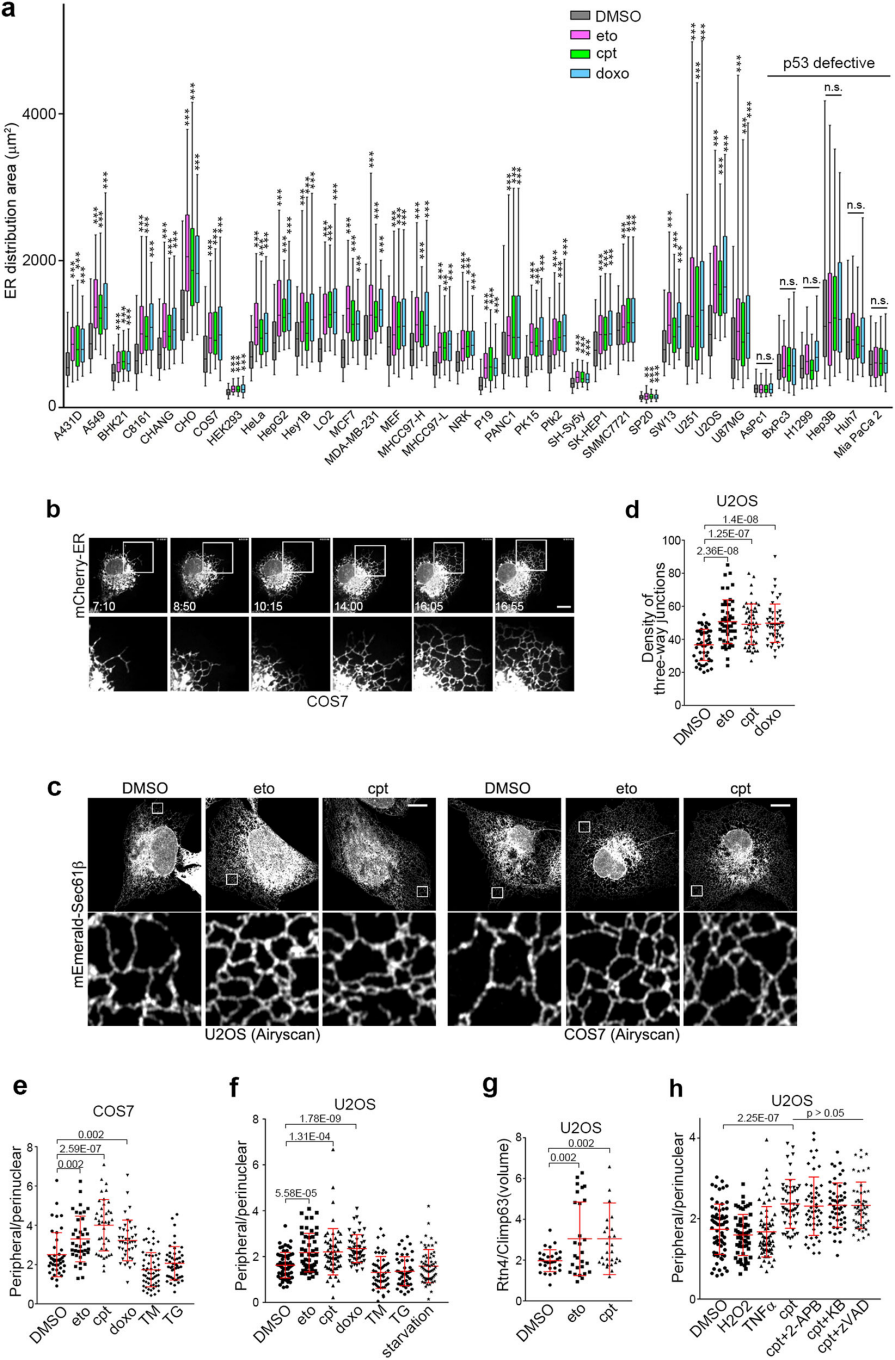
Tubular ER Extension in Response to DNA Damage. **a** Except for HEK293 cells, the indicated cells were treated with DMSO, 50 μM eto, 1 μM cpt or 1 μM doxo for 16 h, and their ER distribution areas were quantified. HEK293 cells were treated with DMSO, 1 μM eto, 100 nM cpt or 100 nM doxo for 16 h. n = 100–300 cells. All cells were transfected with ER lumen-localized mCherry (mCherry-ER). **b** Time-lapse images of a representative COS7 cell transfected with mCherry-ER and treated with 50 μM eto. The indicated times are hour:minute. Boxed areas are magnified below. Scale bar, 10 μm. **c** Representative Airyscan images of mEmerald-Sec61β in U2OS and COS7 cells treated with DMSO, 50 μM eto or 1 μM cpt for 16 h. Boxed regions are magnified. Scale bars, 10 μm. **d** Density of three-way junctions per 200 μm^2^ of U2OS cells transfected with mCherry-ER and treated with DMSO, 50 μM eto, 1 μM cpt or 1 μM doxo for 16 h. **e** Distribution ratio of peripheral/perinuclear ER in COS7 cells exposed to DMSO, 50 μM eto, 1 μM cpt, 1 μM doxo, 2 μM TM or 100 nM TG for 16 h. **f** Distribution ratio of peripheral/perinuclear ER in U2OS cells treated with DMSO, 50 μM eto, 1 μM cpt, 1 μM doxo, 2 μM TM, 100 nM TG, or cultured in medium depleted with amino acids (starvation) for 16 h. **g** Statistical analysis of the volume ratio of fluorescently co-immunolabeled Rtn4 (tubular ER) to Climp63 (ER sheets) in U2OS cells treated with DMSO, 50 μM eto or 1 μM cpt for 16 h. **h** Distribution ratio of peripheral/perinuclear ER in U2OS cells treated with the indicated agents (DMSO, 1 mM H_2_O_2_, 200 ng/mL TNFα, 1 μM cpt, 100 μM 2-APB, 10 μM KB-R7943 or 50 μM z-VAD-FMK) for 16 h

To determine whether DNA damage affects ER morphology, we assessed changes of the total ER distribution area in various mammalian cell lines by expressing ER-lumen localized mCherry (mCherry-ER) and treating the cells with the DNA damaging drugs etoposide (eto), camptothecin (cpt) or doxorubicin (doxo). In 30 of 36 mammalian cell lines tested, exposure to DNA damaging drugs dramatically increased the total ER distribution area (Fig. 1a), indicating that this is a common characteristic of the DNA damage response in mammalian cells. We subsequently focused on COS7 and U2OS cells, which are widely used to study ER morphology.^9,37^ After eto treatment, the ER -- especially the tubular ER -- began to extend after approximately 8 h, reaching its maximum at approximately 15 h in COS7 cells (Fig. 1b; Supplementary Information, Videos S1 and S2). Similar results were observed in both COS7 and U2OS cells treated with cpt or doxo (Supplementary Information, Figure S1a). Airyscan super-resolution microscopy in U2OS cells expressing mEmerald-Sec61β and treated with eto or cpt confirmed the peripheral tubular ER extension after DNA damage (Fig. 1c). Thus, we conclude that DNA damage induces peripheral tubular ER extension.

We then analyzed the morphological changes of the ER in detail. The density of three-way-junctions of the peripheral tubular ER increased significantly after treatment with DNA damaging drugs (Fig. 1b–d). ER sheets are mainly condensed in the perinuclear region while the tubular ER spread outs to the cell periphery.^6^ Considering that it is very difficult to distinguish exactly between sheets and tubules especially in the perinuclear region with current imaging resolution,^38^ we adopted a previously reported threshold method with some modification.^39^ Thresholded mCherry-ER is colocalized well with two ER sheet markers, Kinectin and TRAPα^6^ (Supplementary Information, Figure S1b), suggesting that this provides a good method for estimating the distribution area of ER sheets. We quantified the distribution area of the nucleus and total ER from the original images, and areas of perinuclear ER sheets from thresholded images, and then determined the relative distribution area of perinuclear ER sheets and peripheral ER tubules (Supplementary Information, Figure S1c). We found that the distribution areas of the nucleus and perinuclear ER increased moderately (Supplementary Information, Figure S1d) whereas the distribution areas of the peripheral ER increased markedly upon DNA damage in U2OS cells (Supplementary Information, Figure S1d). To minimize any bias in the quantification, we adopted the distribution ratio between the peripheral and perinuclear (peripheral/perinuclear) as an indicator of tubular ER extension in subsequent studies (Supplementary Information, Figure S1c). Indeed, the peripheral/perinuclear ER ratio increased significantly after treatment with DNA damaging drugs both in COS7 and U2OS cells (Fig. 1e, f).

To further confirm the extension of tubular ER upon DNA damage, we fluorescently co-immunolabeled Rtn4 and Climp63, markers of tubular and sheet ER, respectively.^6,7^ After deconvolution and 3D-reconstruction, we analyzed the volumetric ratio of Rtn4-positive objects to Climp63-positive objects, representing the volumetric ratio of tubular ER to ER sheets. The volumetric ratio of Rtn4/Climp63 also increased significantly after eto- or cpt-treatment (Fig. 1g; Supplementary Information, Figure S1e), confirming that DNA damage induces tubular ER extension.

We then analyzed protein levels of several ER-localized proteins from various pathways. Of these, the Ca^2+^ transporter SERCA2, lipid synthase MOGAT2, and the protein folding chaperone calnexin did not increase upon DNA damage whereas CEPT1 and BiP decreased (Supplementary Information, Figure S1f). Thus, the observed tubular ER extension is not due to an increase in general ER content.

To exclude the possibility that ER extension upon DNA damage is a secondary effect of changes in cell shape, we analyzed the volume occupied by ER and the whole cell by 3D-reconstruction of mCherry-ER and GFP (as an indicator of the whole cell) using 3D structured illumination microscopy (3D-SIM) (Supplementary Information, Figure S1g-i). The total cell volume did not increase (Supplementary Information, Figure S1h) whereas the ER volume increased significantly (Supplementary Information, Figure S1i). Moreover, treating cells with the actin-depolymerizing agent cytochalasin B did not block the DNA damage-induced change in ER distribution (Supplementary Information, Figure S1j and k). The polymerization status of microtubules also was not changed after eto treatment (Supplementary Information, Figure S1l). Therefore, the DNA damage-induced morphological changes of the ER are not likely to be secondary effects of cell shape changes.

Next, to exclude the possibility that the tubular ER extensions are secondary effects of DNA damage-induced apoptosis, we treated cells with cpt together with apoptosis inhibitors targeting different steps of apoptosis: the inositol trisphosphate receptor (IP3R) inhibitor 2-aminoethoxydiphenyl borate (2-APB), the mitochondrial uniporter inhibitor KB-R7943, and the caspase inhibitor zVAD-FMK. We found that the peripheral/perinuclear ratio of ER still increased upon co-treatment with these apoptosis inhibitors (Fig. 1h), suggesting that tubular ER extension is an early event in DNA damage-induced apoptosis that occurs before Ca^2+^ release from the ER.

We then determined whether tubular ER extension is specific to DNA damage by testing several other apoptosis-inducing treatments, including amino acid depletion, H_2_O_2_, ER stress-inducing tunicamycin (TM) and thapsigargin (TG), and the extrinsic apoptosis ligand TNFα. None of these treatments induced an increase in the peripheral/perinuclear ER ratio (Fig. 1e, f, h), suggesting that tubular ER extension is a specific feature of DNA damage.

### DNA damage triggers tubular ER extension through p53

**Fig. 2:**
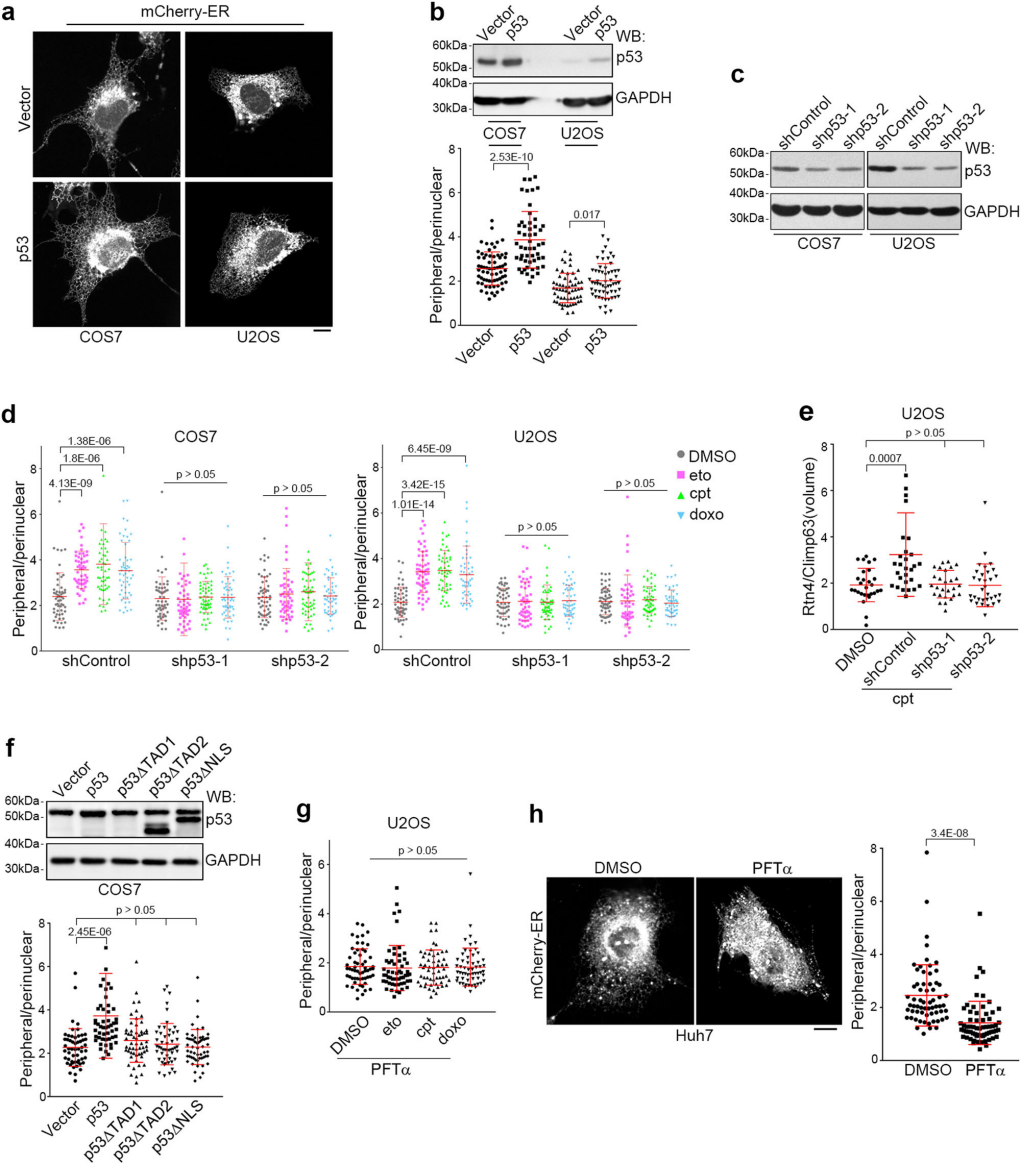
p53 Mediates DNA Damage-induced Tubular ER Extension. **a** Representative images of the ER in COS7 and U2OS cells overexpressing control (vector) or p53. Scale bar, 10 μm. **b** Corresponding western blot (WB) analysis (upper) and the distribution ratio of peripheral/perinuclear ER (lower) in COS7 and U2OS cells overexpressing control or p53. GAPDH served as a loading control. **c** Knockdown efficiency of p53 in COS7 and U2OS cells transfected with control (shControl) or p53 (shp53) shRNA and analyzed by western blot. **d** Distribution ratio of peripheral/perinuclear ER in control or p53 shRNA-transfected COS7 and U2OS cells exposed to DMSO, 50 μM eto, 1 μM cpt or 1 μM doxo for 16 h. **e** Volume ratio of fluorescently co-immunolabeled Rtn4/Climp63 in U2OS cells transfected with control or p53 shRNAs and treated with DMSO or 1 μM cpt for 16 h. **f** Corresponding western blot analysis (upper) and distribution ratio of peripheral/perinuclear ER (lower) in COS7 cells overexpressing p53 or its mutants. Note that the anti-p53 antibody recognizes the TAD1 region of p53, thus is unable to detect p53ΔTAD1. **g** Distribution ratio of peripheral/perinuclear ER in U2OS cells treated with 20 μM PFTα together with DMSO, 50 μM eto, 1 μM cpt or 1 μM doxo for 16 h. **h** Representative images (left) and statistical analysis (right) of the distribution ratio of peripheral/perinuclear ER in Huh7 cells treated with DMSO or 20 μM PFTα. Scale bar, 10 μm

We noticed that among all the tested cell lines, only p53-defective cell lines such as p53-*null* Hep3B^40^ and p53 constitutively active (thus unable to respond to DNA damage) Huh7^41^ cells failed to undergo tubular ER extension after treatment with DNA damage drugs (Fig. 1a; Supplementary Information, Figure S2a and b), suggesting that DNA damage-induced tubular ER extension is p53-dependent. As expected, p53 overexpression led to significant extension of tubular ER (Fig. 2a, b). On the other hand, knockdown of p53 eliminated tubular ER extension upon exposure to the DNA damaging drugs (Fig. 2c–e), indicating that p53 mediates DNA damage-induced extension of tubular ER. To determine whether p53 transcriptional activation is involved, we used p53 mutants lacking either the transactivation domain (p53ΔTAD1/2) or the nuclear localization signal (p53ΔNLS). None of these mutants promoted tubular ER extension (Fig. 2f). Moreover, treatment with pifithrin-α (PFTα), an agent that inhibits p53-mediated transcription,^42^ also blocked DNA damage-induced tubular ER extension (Fig. 2g). The inhibition of p53 by PFTα in Huh7 cells, which contain hyperactive p53, led to a dramatic decrease of the peripheral/perinuclear ratio of the ER (Fig. 2h). Furthermore, COS7 cells that expressed the SV40 large T antigen and are considered to have limited p53 activity,^43^ required much higher doses of DNA damaging drugs (100× that of eto and 25× that of cpt) than required by U2OS cells to exhibit significant changes in ER morphology (Supplementary Information, Figure S2c-j). To further determine the role of p53 in DNA damage-induced ER extension, we re-introduced wild-type p53 or two oncogenic p53 mutants (R248W and R273H), which are defective in transcriptional ability,^44^ into p53 knockout HCT116 cells. As expected, wild-type but not mutant p53 rescued the ER extension response upon DNA damage (Supplementary Information, Figure S2k). Together, these data illustrate that p53-mediated transactivation is required for DNA damage-induced tubular ER extension.

### REEP1/2 are downstream targets of p53

**Fig. 3:**
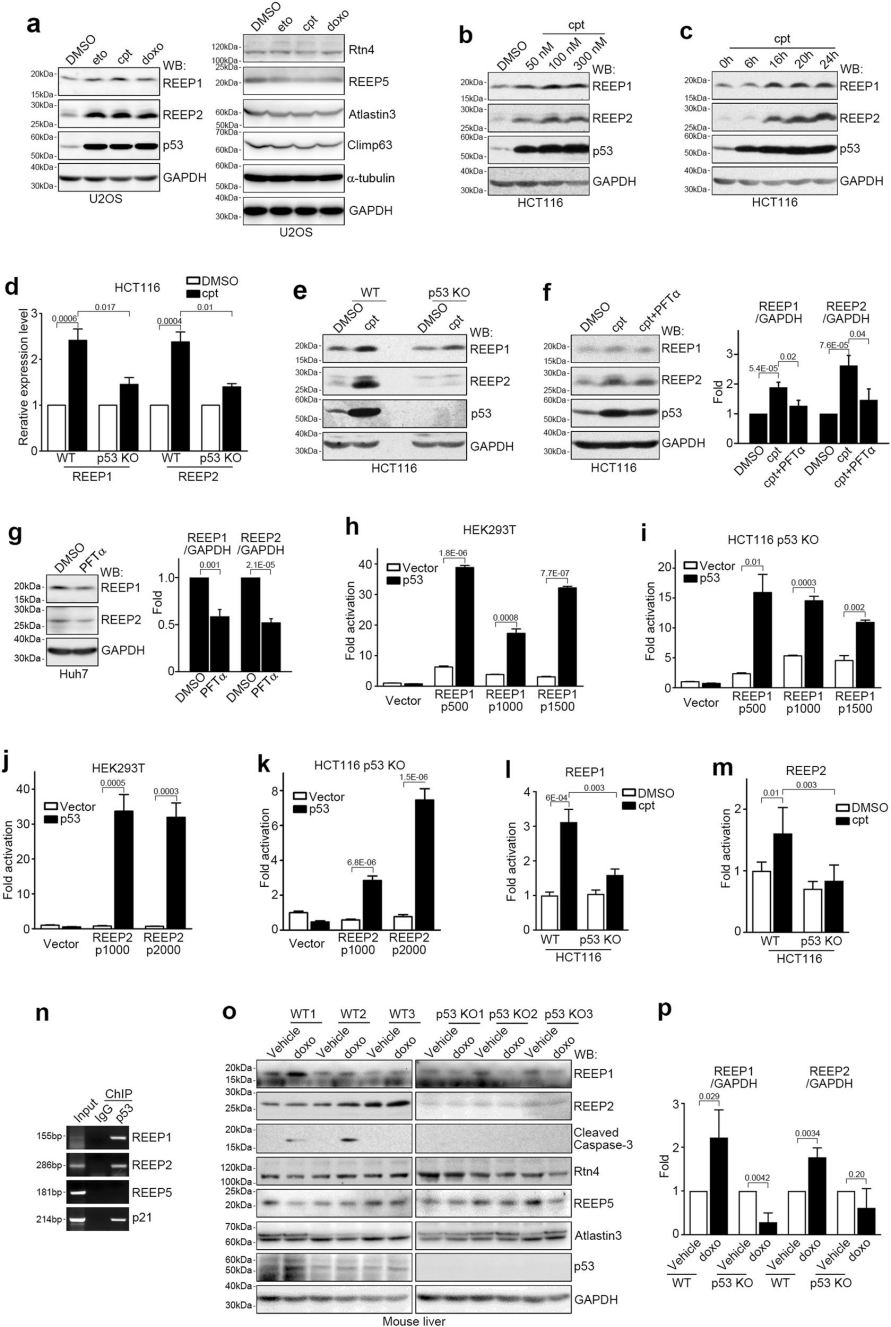
REEP1/2 Are Novel p53 Transcriptional Targets. **a** Western blot (WB) analysis of lysates of U2OS cells treated with DMSO, 50 μM eto, 1 μM cpt or 1 μM doxo for 16 h. GAPDH served as a loading control. **b**, **c** Western blot analysis of lysates from HCT116 cells treated with the indicated doses of cpt (**b**) or 100 nM cpt for the indicated times (**c**). **d** Wild-type (WT) or p53 knockout HCT116 cells were treated with DMSO or 100 nM cpt for 16 h, and the REEP1/2 mRNA level was analyzed by quantitative real-time PCR. n = 3. **e** Wild-type or p53 knockout (KO) HCT116 cells were treated with DMSO or 100 nM cpt for 16 h, and lysates were analyzed by western blot. **f** Western blot analysis of lysates from HCT116 cells treated with DMSO, 100 nM cpt or 100 nM cpt and 100 μM PFTα for 16 h. The results of the band intensity analysis are shown in the right panel. n = 13 independent experiments. **g** Western blot analysis of lysates from Huh7 cells treated with DMSO or 50 μM PFTα for 36 h. Results of the band intensity analysis are shown in the right panel. n = 4 independent experiments. **h**–**k** Luciferase assay of HEK293T (**h**, **j**) or p53 knockout HCT116 (**i**, **k**) cells co-transfected with p53 and REEP1 (**h**, **i**) or REEP2 (**j**, **k**) promoter reporter plasmids. The number indicates base pairs upstream of the transcription initiation site. n = 3. **l**, **m** Wild-type or p53 knockout HCT116 cells were transfected with a REEP1 (p500) (**l**) or REEP2 (p1000) (**m**) promoter reporter plasmid, treated with 100 nM cpt for 16 h, and subjected to the luciferase assay. n = 3. **n** Chromosome immunoprecipitation (ChIP) assay of HEK293 cells using normal IgG or anti-p53 antibody. **o**, **p** Western blots (**o**) and corresponding quantifications (**p**) of lysates from livers of wild-type or p53 knockout mice injected with 20 mg/kg doxo with the indicated antibodies

To identify the downstream regulators of p53 in DNA damage-induced tubular ER extension, we examined relative mRNA expression levels of 24 reported ER-shaping proteins after inducing DNA damage. Of those tested, only the mRNA levels of REEP1 and REEP2 increased significantly after cpt treatment in U2OS cells (Supplementary Information, Figure S2l). We further confirmed that the mRNA levels of REEP1/2, but not REEP5, Rtn4, Atlastin3 or Climp63, were upregulated after treatment with eto or cpt, but not the ER stress-inducing drug TM (Supplementary Information, Figure S2m). Likewise, protein levels of REEP1/2 -- but not REEP5, Rtn4, Atlastin3 or Climp63 -- also increased dramatically after being exposed to DNA damaging drugs but not to other apoptosis-inducing agents including TM, TG, H_2_O_2_, staurosporine (STS), and puromycin (Fig. 3a; Supplementary Information, Figure S2n). Moreover, cpt upregulated protein levels of REEP1/2 in a dose- and time-dependent manner (Fig. 3b, c). Collectively, these data indicate that REEP1/2 are selectively upregulated in response to DNA damage.

We then investigated whether REEP1/2 upregulation is dependent on p53. Overexpression of p53 in HEK293 cells resulted in increased mRNA levels of REEP1/2, but not REEP5, Rtn4, Atlastin3 or Climp63 (Supplementary Information, Figure S2o). Treatment with cpt failed to upregulate REEP1/2 mRNA levels in p53-*null* Hep3B cells (Supplementary Information, Figure S2p). Compared with wild-type HCT116 cells, p53 knockout HCT116 cells also showed a much weaker response to cpt treatment for both REEP1/2 mRNA (Fig. 3d) and protein levels (Fig. 3e). Addition of the p53 inhibitor PFTα suppressed cpt-induced REEP1/2 upregulation (Fig. 3f). It also led to decreased REEP1/2 protein levels in p53-hyperactive Huh7 cells (Fig. 3g). Furthermore, re-introduction of wild-type but not oncogenic mutant forms of p53 (R248W and R273H) rescued the DNA damage-induced increase in REEP1/2 protein levels (Supplementary Information, Figure S2q). Together, these results indicate that REEP1/2 are downstream targets of p53.

Next, we asked whether REEP1/2 are direct transcriptional targets of p53. Results from a luciferase assay showed that both REEP1 and REEP2 promoters are significantly activated by p53 overexpression (Fig. 3h–k). Treatment with cpt also dramatically enhanced REEP1/2 promoter activity in wild-type but not p53 knockout HCT116 cells (Fig. 3l, m). Chromosome immunoprecipitation further verified that endogenous p53 bound to the REEP1/2 promoters (Fig. 3n). Together, these data suggest that REEP1/2 are direct downstream targets of p53.

To test whether REEP1/2 are also p53 downstream proteins in vivo, we intraperitoneally injected doxo to wild-type or p53 knockout mice and detected the protein levels of REEP1/2 in the liver. As expected, protein levels of REEP1/2 but not REEP5, Rtn4, Atlastin3 or Climp63, were upregulated in wild-type mice after doxo treatment (Fig. 3o); on the contrary, in p53 knockout mice, doxo treatment failed to increase protein levels of REEP1/2 and also suppressed apoptosis (Fig. 3o, p), suggesting that DNA damage activates REEP1/2 expression through p53 in vivo.

### REEP1/2 are required for DNA damage-induced tubular ER extension

**Fig. 4:**
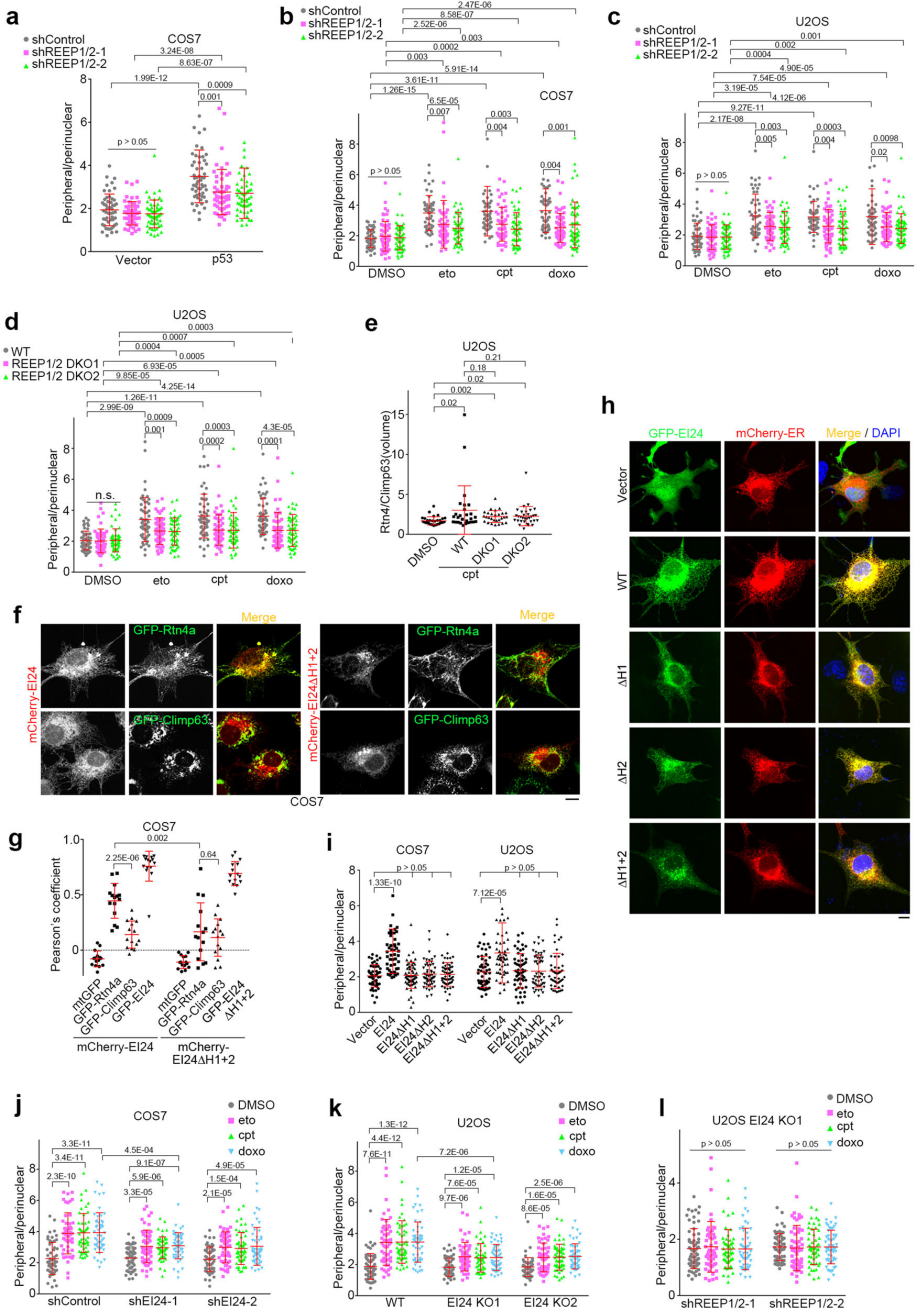
REEP1/2 and EI24 are Responsible for DNA Damage-induced Tubular ER Extension. **a** Distribution ratio of peripheral/perinuclear ER in COS7 cells overexpressing control (vector) or p53, together with control (shControl) or REEP1/2 shRNAs (shREEP1/2). shREEP1/2 indicates co-transfection of shREEP1 and shREEP2. **b**, **c** Distribution ratio of peripheral/perinuclear ER in COS7 (**b**) or U2OS (**c**) cells transfected with control or REEP1/2 shRNAs and treated with DMSO, 50 μM eto, 1 μM cpt or 1 μM doxo for 16 h. **d** Distribution ratio of peripheral/perinuclear ER in wild-type (WT) or REEP1/2 double knockout (DKO) U2OS cells treated with DMSO, 50 μM eto, 1 μM cpt or 1 μM doxo for 16 h. **e** ER volume ratio of fluorescently co-immunolabeled Rtn4/Climp63 in wild-type or REEP1/2 double knockout U2OS cells treated with DMSO or 1 μM cpt. **f** Representative images of COS7 cells co-overexpressing mCherry-EI24 or mCherry-EI24ΔH1 + 2 and either GFP-Rtn4a (upper panel) or GFP-Climp63 (lower panel). Scale bar, 10 μm. **g** Co-localization analysis of EI24 or EI24ΔH1 + 2 with the indicated markers as **f**. **h** Representative images of COS7 cells transfected with mCherry-ER (red) and GFP-tagged either wild-type or truncated EI24 constructs (green). Scale bar, 10 μm. DNA was stained with DAPI (blue). **i** Distribution ratio of peripheral/perinuclear ER in COS7 and U2OS cells transfected with wild-type or truncated EI24 constructs (n ≥ 50 cells). **j** Distribution ratio of peripheral/perinuclear ER in COS7 cells transfected with either control or EI24 shRNA and exposed to DMSO, 50 μM eto, 1 μM cpt or 1 μM doxo for 16 h. **k** Distribution ratio of peripheral/perinuclear ER in wild-type and EI24-knockout U2OS cells treated with DMSO, 50 μM eto, 1 μM cpt or 1 μM doxo for 16 h. **l** Distribution ratio of peripheral/perinuclear ER in EI24-knockout U2OS cells transfected with either control or REEP1/2 shRNA and treated with DMSO, 50 μM eto, 1 μM cpt or 1 μM doxo for 16 h

Consistent with previous reports that REEP1/2 are ER-shaping proteins,^8,12^ REEP1/2 overexpression significantly promoted tubular ER formation in cell lines (Supplementary Information, Figure S3a). We then knocked down REEP1/2 by co-transfecting REEP1 and REEP2 shRNAs (Supplementary Information, Figure S3b). Knockdown of REEP1/2 resulted in a decreased peripheral/perinuclear ratio in p53-hyperactive Huh7 cells (Supplementary Information, Figure S3c) and weakened the effects of p53 overexpression on ER morphology in COS7 cells (Fig. 4a), illustrating that p53 promotes tubular ER extension via REEP1/2. Moreover, knockdown of REEP1/2 significantly weakened the tubular ER extension induced by DNA damaging drugs (Fig. 4b, c), suggesting that REEP1/2 are responsible for DNA damage-induced tubular ER extension. We further prepared REEP1/2 double-knockout U2OS cells using CRISPR/Cas9 approaches^45^ (Supplementary Information, Figure S3d). Knockout of REEP1/2 also reduced, but did not eliminate, the extension of tubular ER after the addition of DNA damaging drugs (Fig. 4d, e), suggesting that other factors might also be involved.

### EI24 promotes tubular ER formation and is required for DNA damage-induced tubular ER extension

To identify additional factors in the p53-mediated change to ER morphology induced by DNA damage, we searched for p53 target ER membrane proteins and noticed EI24, a transcriptional target of p53.^23,28^ Bioinformatic analyses showed that EI24 contains two potential intramembrane hairpin regions (Supplementary Information, Figure S3e). mCherry-EI24 colocalized well with the tubular ER-marker GFP-Rtn4a but to a lesser extent with GFP-Climp63, a marker of ER sheets (Fig. 4f, g). In contrast, the hairpin region deletion mutant EI24ΔH1 + 2 barely colocalized with either of these two markers (Fig. 4f, g). This suggests that EI24 localizes largely at the tubular ER and that the hydrophobic hairpin regions are important for its tubular ER localization. Overexpression of EI24 significantly facilitated the extension of the tubular ER (Fig. 4h, i), whereas deletion of either hairpin region of EI24 diminished this effect (Fig. 4h, i), despite retaining ER localization (Fig. 4h). Thus, both hairpin regions are required for EI24 to promote tubular ER formation.

ER-shaping proteins tend to interact with each other via their intramembrane hairpin regions.^8,9^ Immunoprecipitation results also showed the association of EI24 with Rtn4a, Lnp1 and REEP1/2 (Supplementary Information, Figure S3f-j), but not Atlastin1 (Supplementary Information, Figure S3k), indicating that EI24 only associates with a subset of ER-shaping proteins. In addition, EI24 that lacked hairpin regions failed to interact with Rtn4a (Supplementary Information, Figure S3l), suggesting that the interaction between EI24 and Rtn4a is mediated by the hairpin regions of EI24.

Consistent with previous reports that EI24 is a protein downstream of p53,^23^ we found that EI24 protein levels increased upon exposure to DNA damaging drugs (Supplementary Information, Figure S3m and n). EI24 knockdown weakened the effects of DNA damage on the extension of tubular ER (Fig. 4j; Supplementary Information, Figure S3o). EI24 knockout also consistently impaired DNA damage-induced extension of tubular ER (Fig. 4k; Supplementary Information, Figure S3p). Intriguingly, knockdown of REEP1/2 in EI24-knockout U2OS cells almost fully blocked tubular ER extension upon DNA damage (Fig. 4l), suggesting that EI24 is a key factor that cooperates with REEP1/2 to promote the extension of tubular ER under DNA damage conditions.

### EI24- and REEP1/2-mediated tubular ER extension is required for DNA damage-induced apoptosis

**Fig. 5:**
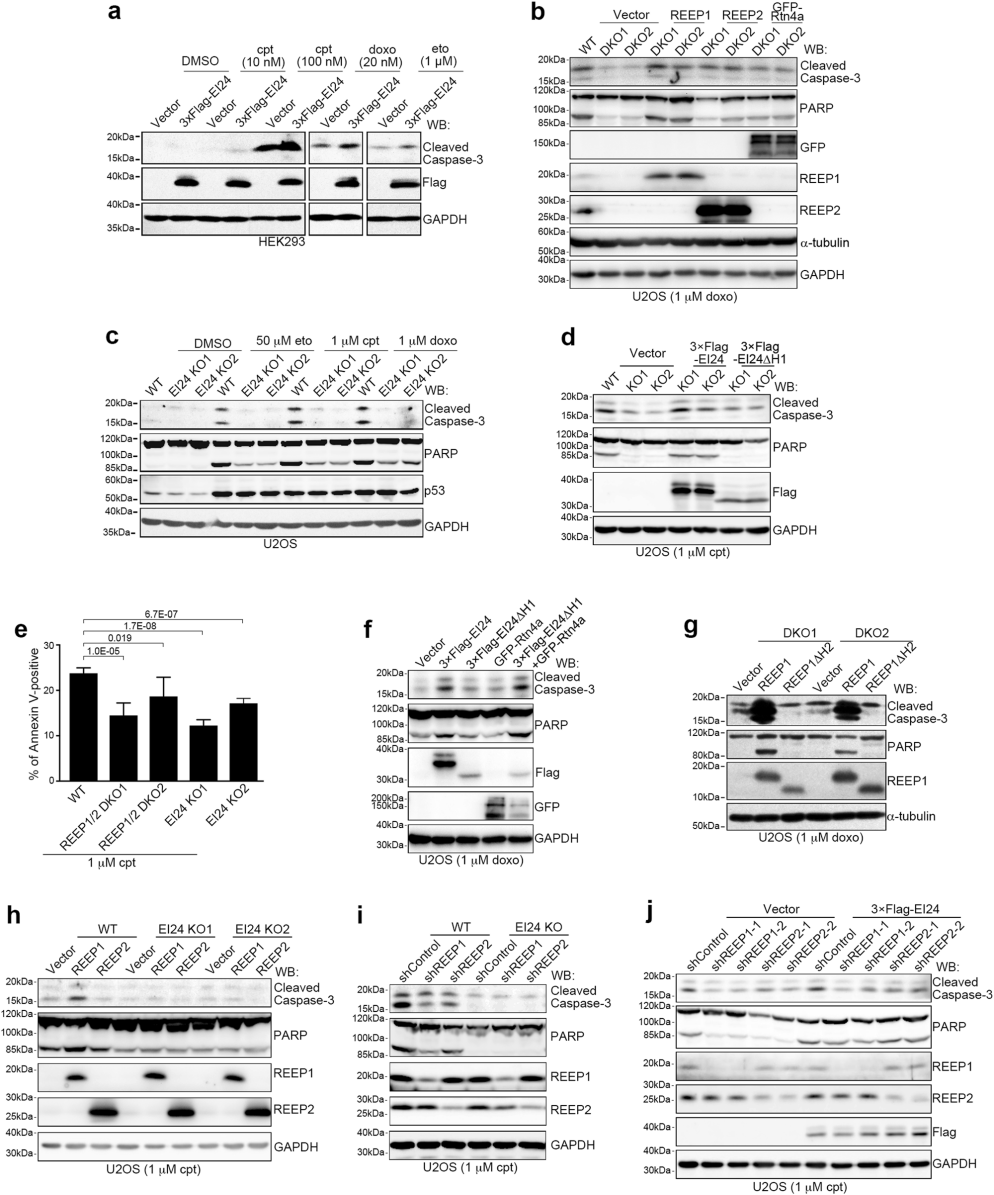
REEP1/2- and EI24-mediated ER Shaping are Required for DNA Damage-induced Apoptosis. **a** Western blot (WB) analysis of lysates from HEK293 cells transfected with the vector or 3× Flag-EI24 and treated with DMSO, cpt, doxo or eto for 12 h. GAPDH served as a loading control. **b** Western blot analysis of lysates from wild-type (WT) or REEP1/2 double knockout (DKO) U2OS cells transfected with the indicated plasmids and treated with 1 μM doxo for 24 h. **c** Western blot analysis of lysates from wild-type and EI24-knockout (KO) U2OS cells treated with DMSO, 50 μM eto, 1 μM cpt or 1 μM doxo for 24 h. **d** Western blot analysis of wild-type and EI24-knockout U2OS cells transfected with 3× Flag-EI24 or 3× Flag-EI24ΔH1 and treated with 1 μM cpt for 24 h. **e** Quantification of flow cytometry data for wild-type, REEP1/2 double knockout (DKO), or EI24 knockout cells positive for Annexin V. Cells were treated with 1 μM cpt for 14 h. **f** Western blot analysis of U2OS cells overexpressing the indicated proteins and treated with 1 μM doxo for 24 h. **g** Western blot analysis of lysates from REEP1/2 double knockout U2OS cells transfected with the vector, REEP1 or REEP1ΔH2 and treated with 1 μM doxo for 24 h. **h** Western blot analysis of wild-type and EI24-knockout U2OS cells transfected with REEP1/2 and treated with 1 μM cpt for 24 h. **i** Western blot analysis of wild-type and EI24-knockout U2OS cells transfected with control (shControl) or REEP1/2 shRNA (shREEP1/2) and treated with 1 μM cpt for 24 h. **j** Western blot analysis of U2OS cells transfected with the indicated shRNA alone or together with 3× Flag-EI24 and treated with 1 μM cpt for 24 h

DNA damage response pathways mainly involve cell cycle arrest, DNA repair and apoptosis.^21^ Flow cytometric analyses showed that REEP1/2 and EI24 did not affect DNA damage-induced cell cycle arrest (Supplementary Information, Figure S4a-d). We also assessed components of DNA damage response pathways and downstream targets of p53 and showed that their protein levels were not affected by REEP1/2 knockdown or knockout (Supplementary Information, Figure S4e-g). However, overexpression of EI24 and REEP1, but not REEP2, enhanced DNA damage-induced apoptosis, as revealed by cleavage of caspase-3 and poly ADP-ribose polymerase (PARP) (Fig. 5a; Supplementary Information, Figure S5a), whereas knockout of both REEP1/2 and EI24 suppressed apoptosis after treatment with DNA damaging drugs and this was rescued by re-expression of REEP1/2 and EI24, respectively (Fig. 5b–d). Knockdown of REEP1/2 or EI24 gave similar results (Supplementary Information, Figure S5b-d). We also used flow cytometry to detect Annexin V-positive cells and showed that they were significantly reduced after knockout of REEP1/2 or EI24 following treatment with cpt (Fig. 5e; Supplementary Information, Figure S5e). Cell survival tests by MTT assay (for NAD(P)H-dependent cellular oxidoreductases active on 3-(4,5-dimethylthiazol-2-yl)-2,5-diphenyltetrazolium bromide) carried out following the overexpression or depletion of REEP1/2 or EI24 showed consistent results (Supplementary Information, Figure S5f-i). Together, these data suggest that REEP1/2 and EI24 are required for DNA damage-induced apoptosis.

Next, we investigated the relationship between REEP1/2- and EI24-mediated ER extension and DNA damage-induced apoptosis. Overexpression of REEP1, REEP2 or Rtn4a in untreated cells did not induce apoptosis (Supplementary Information, Figure S5j). Moreover, the overexpression of Rtn4a also did not promote apoptosis in cells treated with the DNA damaging drug doxo (Fig. 5f). This suggests that tubular ER extension itself is not sufficient to induce apoptosis. However, overexpression of Rtn4a rescued DNA damage-induced apoptosis in REEP1/2-deficient cells (Fig. 5b; Supplementary Information, Figure S5b). Moreover, as expected, overexpression of REEP1ΔH2, which lacks its second intramembrane hairpin domain and is unable to induce tubular ER extension (Supplementary Information, Figure S5k), neither promoted apoptosis (Supplementary Information, Figure S5l) nor rescued the impaired apoptosis of REEP1/2 knockout cells upon treatment with DNA damaging drugs (Fig. 5g). Similarly, overexpression of EI24ΔH1/2/1 + 2 failed to increase DNA damage-induced apoptosis (Supplementary Information, Figure S5m). EI24ΔH1 did not rescue the impaired apoptosis in EI24-deleted cells either (Fig. 5d; Supplementary Information, Figure S5d) whereas co-transfection of Rtn4a with EI24ΔH1 greatly enhanced apoptosis (Fig. 5f). Together, these data suggest that tubular ER extension is required for DNA damage-induced apoptosis and is necessary for REEP1 and EI24 to promote DNA damage-induced apoptosis.

We further examined the functional differences between EI24 and REEP1/2 in DNA damage-induced apoptosis and found that neither overexpression nor knockdown of REEP1/2 affected DNA damage-induced apoptosis in EI24-knockout cells (Fig. 5h, i). In contrast, overexpression of EI24 in REEP1/2 knockdown cells still promoted DNA damage-induced apoptosis (Fig. 5j). Together, these results suggest that REEP1 and REEP2 act as accessory factors to EI24 in promoting DNA damage-induced apoptosis. Therefore, we focused on EI24 in subsequent studies.

We next investigated whether EI24 affects other apoptotic pathways. Interestingly, EI24 overexpression inhibited the induction of apoptosis induced by the ER stress-inducing drugs TM and TG, the extrinsic apoptosis ligand TNFα, puromycin, G418 or the ATP-competitive kinase inhibitor Staurosporine (STS; Supplementary Information, Figure S5n). Consistently, EI24 knockout promoted the induction of apoptosis induced by these stimuli (Supplementary Information, Figure S5o). Moreover, even when anti-apoptotic Bcl-2^46^ is overexpressed, EI24 still promoted DNA damage-induced apoptosis (Supplementary Information, Figure S5p). Therefore, these data suggest that EI24 plays a unique role in DNA damage-induced apoptosis that seems to be independent of canonical Bcl-2 regulation.

### Pathological mutants of EI24 fail to promote tubular ER extension and apoptosis

**Fig. 6:**
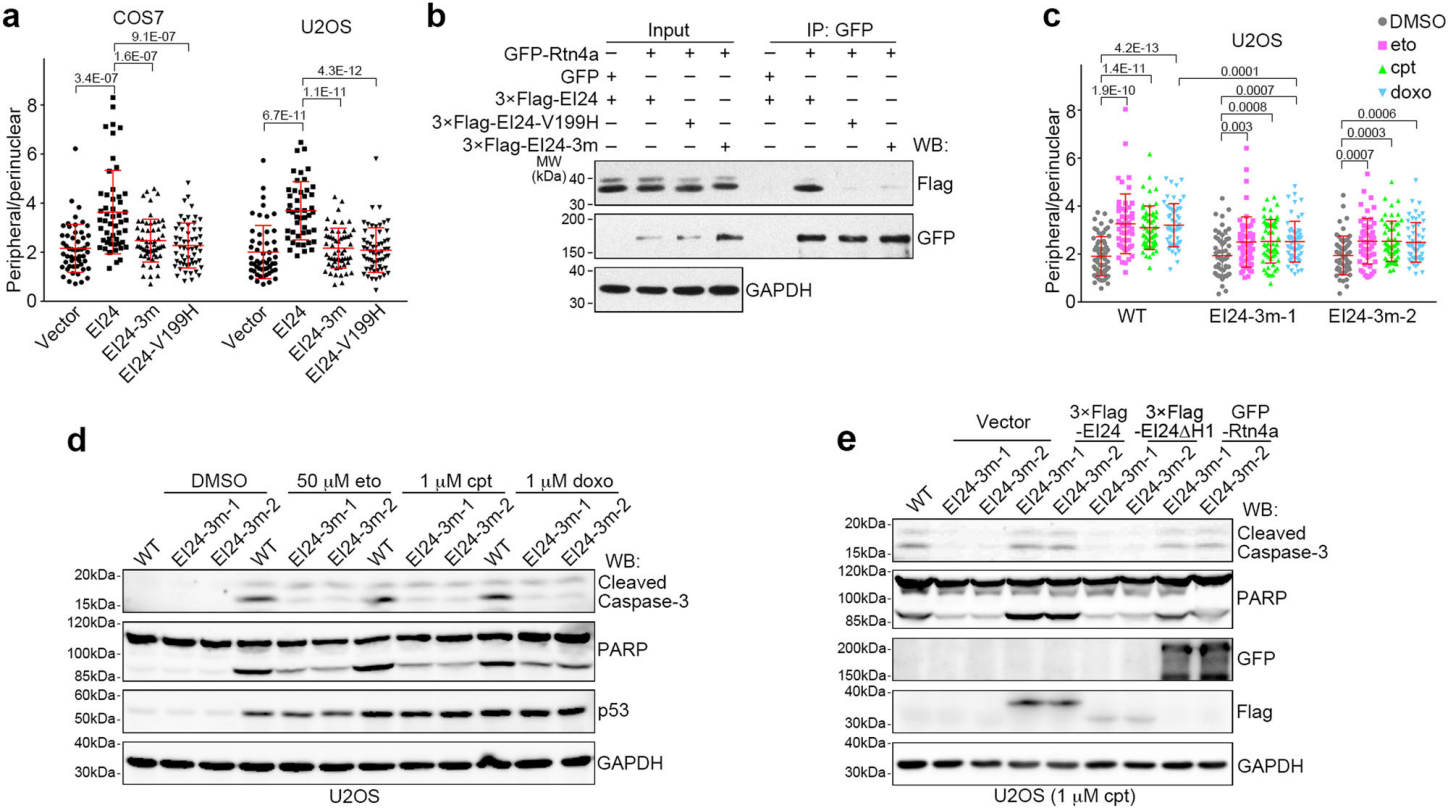
Cancer Mutation in EI24 Failed to Promote DNA Damage-induced Tubular ER Extension and Apoptosis. **a** Distribution ratio of peripheral/perinuclear ER in COS7 and U2OS cells transfected with either EI24 or its point mutants. 3 m represents P195T-I196D-H197Y (n ≥ 50 cells). GAPDH served as a loading control. **b** Immunoprecipitation (IP) assay of GFP-Rtn4a with either 3× Flag-EI24 or its point mutants. **c** Distribution ratio of peripheral/perinuclear ER in wild-type and EI24-3m U2OS cells treated with DMSO, 50 μM eto, 1 μM cpt or 1 μM doxo for 16 h (n ≥ 50 cells). **d** Western blot (WB) analysis of wild-type and EI24-3m U2OS cells treated with DMSO, 50 μM eto, 1 μM cpt, or 1 μM doxo for 24 h. **e** Wild-type and EI24-3m U2OS cells were transfected with the indicated plasmids, treated with 1 μM cpt for 24 h, and analyzed by Western blot

EI24 is frequently lost or mutated in various carcinomas.^24–27^ We noticed that several previously identified breast-cancer-related pathological point mutations of EI24 -- EI24-P195T-I196D-H197Y (hereafter referred to as EI24-3m) and EI24-V199H^24^ -- are both located in the middle of the first intramembrane hairpin region of EI24 (Supplementary Information, Figure S3e). We speculated that these missense mutations might disrupt the function of EI24 in tubular ER formation, thus impairing apoptosis upon genome instability, which could allow tumor formation. Indeed, neither EI24-3m nor EI24-V199H promoted tubular ER extension after overexpression (Fig. 6a; Supplementary Information, Figure S6a). They also did not associate with Rtn4a (Fig. 6b). This suggests that P195-I196-H197 and V199 of EI24 are important for promoting tubular ER formation. We next prepared endogenous EI24-3m mutant U2OS cells (Supplementary Information, Figure S6b) by gene editing using the CRISPR/Cas9 system.^45^ Similar to the effects in EI24 knockout cells (Fig. 4k), DNA damaging drugs induced weaker tubular ER extension in EI24-3m cells compared with wild-type cells (Fig. 6c).

As expected, overexpression of either EI24-3m or EI24-V199H failed to promote DNA damage-induced apoptosis (Supplementary Information, Figure S6c). Consistently, the endogenous EI24-3m mutation significantly impaired apoptosis induced by DNA damaging drugs but not by TM, H_2_O_2_, STS, or puromycin (Fig. 6d and Supplementary Information, Figure S6d). MTT assays further confirmed that the EI24-3m mutations promoted cell survival under conditions of DNA damage (Supplementary Information, Figure S6e). Moreover, in EI24-3m cells, overexpression of the ER-shaping protein Rtn4a was sufficient to rescue impaired apoptosis to an extent similar to that of wild-type EI24 (Fig. 6e), suggesting that the observed inhibition of apoptosis is caused by failure in tubular ER extension.

### EI24 associates with and depends upon VDAC2 to promote DNA damage-induced apoptosis

**Fig. 7:**
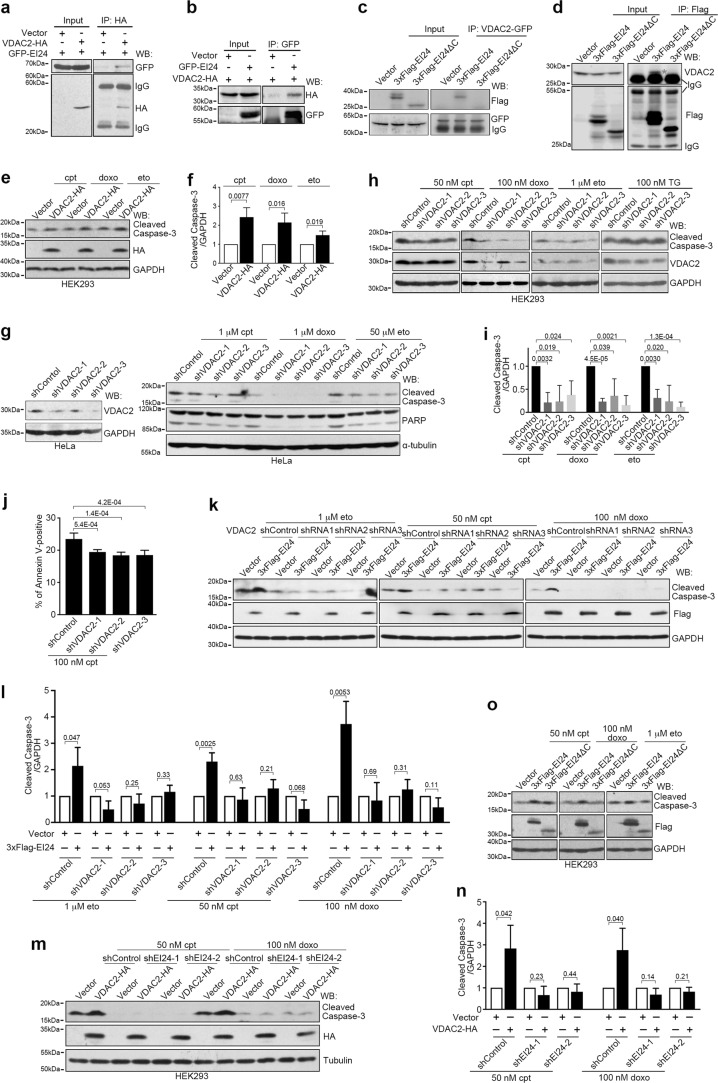
EI24 Associates with VDAC2 and Regulates DNA Damage-induced Apoptosis by VDAC2. **a**, **b** HEK293T cells co-transfected with VDAC2-HA and GFP-EI24 were subjected to immunoprecipitation (IP) assays and western blot (WB) analysis with the indicated antibodies. **c** HEK293T cells co-transfected with VDAC2-GFP and the vector, 3× Flag-EI24 or 3× Flag-EI24ΔC were subjected to immunoprecipitation and western blot analysis with the indicated antibodies. **d** HEK293T cells transfected with the vector, 3× Flag-EI24 or 3× Flag-EI24ΔC were subjected to immunoprecipitation and western blot analysis with the indicated antibodies. **e** Western blot analysis of lysates from HEK293 cells transfected with either the control vector or VDAC2-HA and treated with 50 nM cpt, 50 nM doxo, 1 μM eto for 12 h. GAPDH served as a loading control. **f** Band intensity analysis of **e**. n = 3 independent experiments. **g** Western blot analysis of lysates from HeLa cells transfected with control (shControl) or VDAC2 shRNA (sh VDAC2) and treated with 1 μM cpt, 1 μM doxo or 50 μM eto for 12 h. Tubulin served as a loading control. **h** Western blot analysis of lysates from HEK293 cells transfected with control or VDAC2 shRNA and treated with 50 nM cpt, 100 nM doxo, 1 μM eto or 100 nM TG for 12 h. **i** Band intensity analysis of **h**. n = 3 independent experiments. **j** Quantification of flow cytometry analysis for VDAC2 shRNA-transfected cells positive for Annexin V. Cells were treated with 100 nM cpt 12 h. **k** Western blot analysis of lysates from HEK293 cells co-transfected with the vector or 3× Flag-EI24 and control or VDAC2 shRNA and treated with 1 μM eto, 50 nM cpt or 100 nM doxo for 12 h. **l** Band intensity analysis of **k**. n = 3 independent experiments. **m** Western blot analysis of lysates from HEK293 cells co-transfected with either the vector or VDAC2-HA and control or EI24 shRNA (shEI24) and treated with 50 nM cpt or 100 nM doxo for 12 h. **n** Band intensity analysis of **m**. n = 3 independent experiments. **o** Western blot analysis of lysates from HEK293 cells transfected with the vector, 3× Flag-EI24 or 3× Flag-EI24ΔC and treated with 50 nM cpt, 100 nM doxo or 1 μM eto for 12 h

To investigate the mechanism underlying the function of EI24 in DNA damage-induced apoptosis, we searched for EI24-interacting proteins using immunoprecipitation and mass spectrometry analysis; this revealed several potential binding partners, including VDAC2 (Supplementary Information, Figure S7a). Immunoprecipitation studies confirmed the interaction between EI24 and VDAC2 (Fig. 7a, b), but not VDAC1 and VDAC3 (Supplementary Information, Figure S7b and c). It also established that the C-terminal region (amino acids 279–340) of EI24 was essential for its interaction with VDAC2 (Fig. 7c, d).

Next, we determined whether VDAC2 is required for EI24-promoted apoptosis. In untreated cells, neither individual overexpression nor co-overexpression of EI24 and VDAC2 induced apoptosis (Supplementary Information, Figure S7d). In drug-treated cells, similar to EI24, VDAC2 overexpression promoted apoptosis induced by DNA damaging drugs (Fig. 7e, f), but not by TM, TNFα or TG (Supplementary Information, Figure S7e). Consistently, VDAC2 knockdown inhibited DNA damage-induced apoptosis, but not TG-induced apoptosis (Fig. 7g–j; Supplementary Information, Figure S7f), while knockdown of VDAC1 did not suppress DNA damage-induced apoptosis (Supplementary Information, Figure S7g). Importantly, knockdown of VDAC2 suppressed the ability of overexpressed EI24 to promote DNA damage-induced apoptosis (Fig. 7k, l), and EI24 knockdown also blocked the effect of VDAC2 overexpression on DNA damage-induced apoptosis (Fig. 7m, n). Furthermore, EI24ΔC, the C-terminal deletion mutant of EI24 (1–278 aa), lost its ability to promote DNA damage-induced apoptosis (Fig. 7o). Therefore, VDAC2 cooperates with EI24 to regulate DNA damage-induced apoptosis.

Interestingly, similar to EI24, the protein levels of VDAC2 increased after treatment with drugs leading to DNA damage (Supplementary Information, Figure S7h). In p53-knockout HCT116 cells, treatment with the DNA damaging drugs did not increase the protein levels of VDAC2 (Supplementary Information, Figure S7i), whereas p53 overexpression resulted in an increased protein level of VDAC2 in HCT116, U2OS and HEK293 cells (Supplementary Information, Figure S7j). However, the mRNA levels of VDAC2 were not affected by p53 overexpression (Supplementary Information, Figure S7k and l), suggesting that VDAC2 is not a transcriptional target of p53.

### DNA damage induces an increase in ER-mitochondria contacts through EI24 and VDAC2

**Fig. 8:**
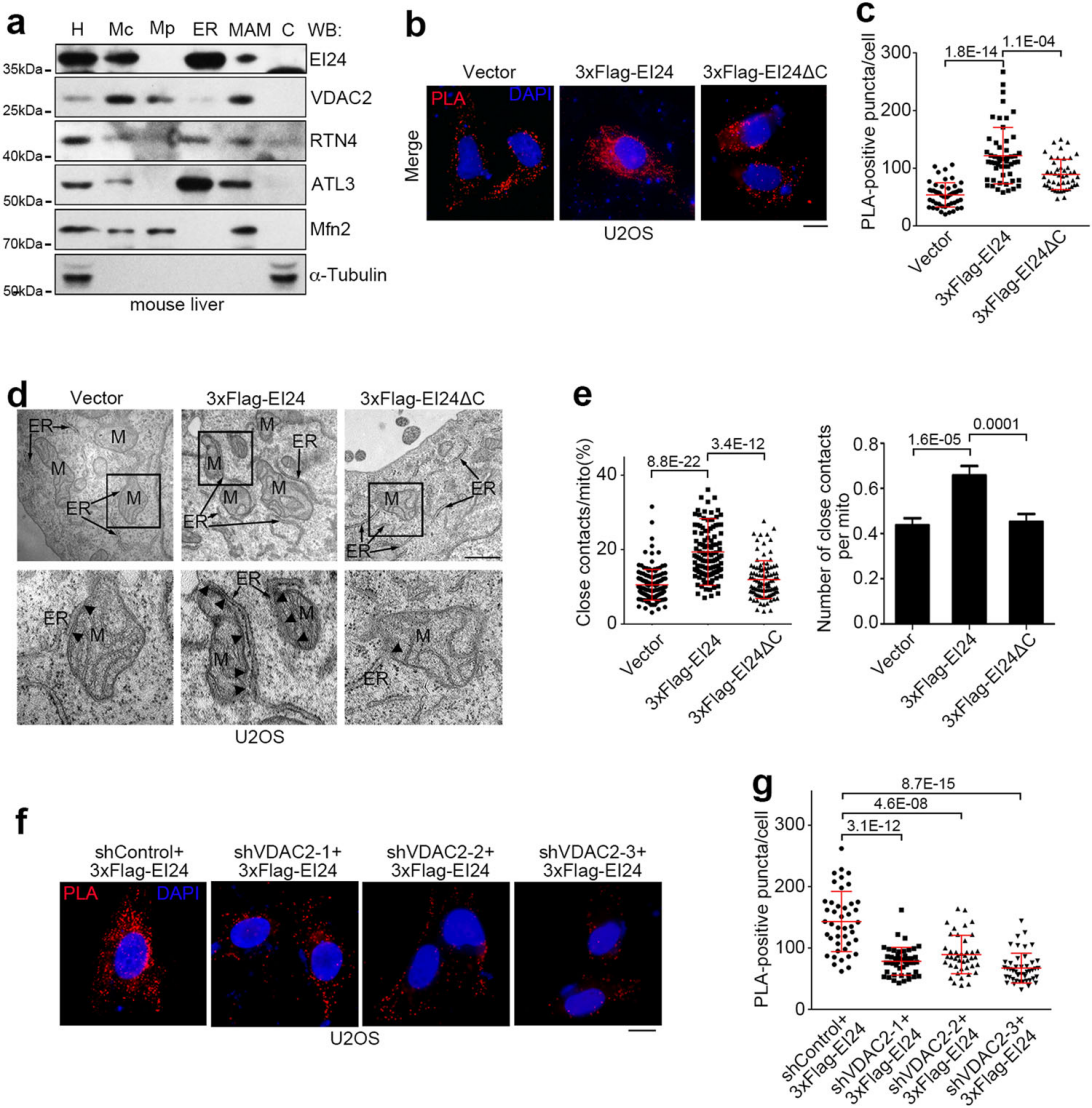
EI24 Promotes ER-mitochondria Contacts by VDAC2. **a** Western blot (WB) analysis of subcellular fractions from mouse liver. H homogenate; Mc crude mitochondrial fraction; Mp pure mitochondrial fraction; ER endoplasmic reticulum; MAM mitochondria-associated membrane; C cytosol. RTN4 Reticulon 4; ATL3 Atlastin 3; Mfn2 mitofusin-2. **b** U2OS cells that stably expressed CFP-ER and overexpressed the vector, 3× Flag-EI24 or 3× Flag-EI24ΔC were subjected to PLA (red) with anti-TOM20 and anti-GFP antibodies. DNA was stained with DAPI (blue). Scale bar, 10 μm. **c** Quantification of the number of PLA-positive puncta/cell in **b**. **d** Representative EM images of U2OS cells transfected with the vector, 3× Flag-EI24 or 3× Flag-EI24ΔC. Boxed areas at the top are magnified at the bottom, and the arrowheads indicate the ER-mitochondria contacts (<30 nm). ER endoplasmic reticulum; M mitochondrion. Scale bar, 500 nm. **e** Quantification of the ratio (%) of ER-mitochondria contacts (<30 nm) to the mitochondrial surface area (left) and the average number of close contacts per mitochondrion (right) in **d** (n = 118–311 mitochondria). **f** U2OS cells that stably expressed CFP-ER were co-transfected with 3× Flag-EI24 and control (shControl) or VDAC2 shRNA (shVDAC2) and subjected to PLA. Scale bar, 10 μm. **g** Quantification of the number of PLA-positive puncta/cell in **f**

Because EI24 is an ER membrane protein and VDAC2 is a mitochondrial outer membrane protein, we speculated that EI24 and VDAC2 might interact at ER-mitochondria contacts. To test this, we analyzed the subcellular distribution of EI24 and VDAC2 in mouse liver using Percoll density-gradient centrifugation.^47^ EI24 and VDAC2 co-existed in the same MAM fractions, along with mitofusin-2 (Mfn2) (Fig. 8a). Then, to examine whether EI24 affects ER-mitochondria contacts, we established U2OS cells that stably expressed CFP-ER, with CFP fused to the ER targeting sequence of yeast UBC6,^48^ and performed a proximity ligation assay (PLA) by probing for the mitochondrial outer membrane protein TOM20 and ER-targeted CFP.

Overexpression of wild-type EI24, but not EI24ΔC, significantly increased the number of red PLA-positive puncta (Fig. 8b, c). Using electron microscopy, we quantified the average ER contact sites of every mitochondria per cell, and the percentage of perimeter of mitochondria that was close (<30 nm) to the ER. The number and the closely apposed area of contacts between ER and mitochondria increased significantly in U2OS cells overexpressing wild-type EI24 but not EI24ΔC (Fig. 8d, e). Importantly, VDAC2 knockdown reversed the increase in the number of ER-mitochondria contacts induced by EI24 overexpression, as revealed by PLA (Fig. 8f, g). Together, these data suggest that EI24 promotes ER-mitochondria contacts via VDAC2.

**Fig. 9:**
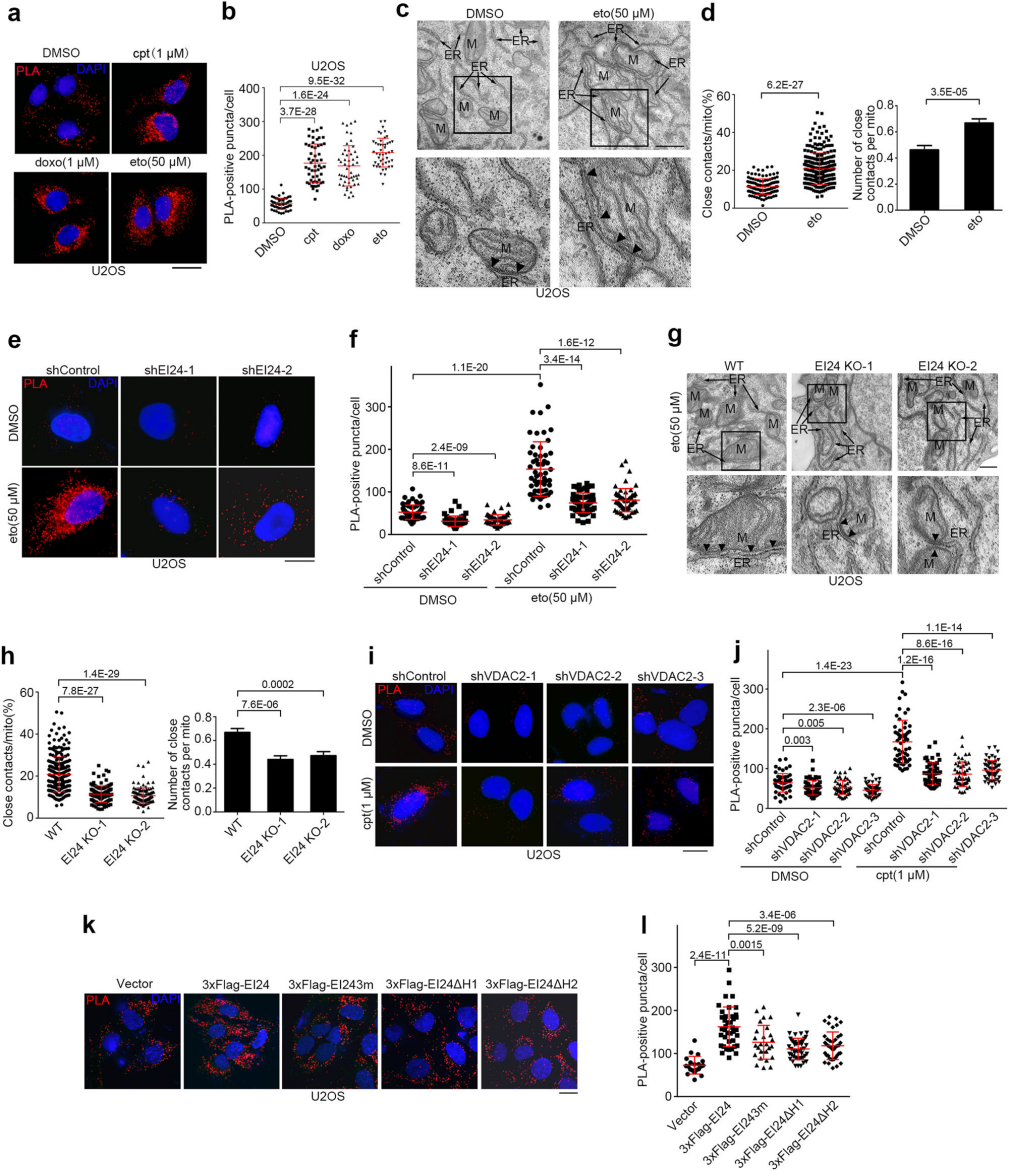
DNA Damage Promotes ER-mitochondria Contacts through EI24 and VDAC2. **a** U2OS cells stably expressing CFP-ER were treated with cpt, doxo or eto for 12 h, and subjected to PLA. Scale bar, 10 μm. **b** Quantification of the number of PLA-positive puncta/cell in **a**. **c** Representative EM images of U2OS cells treated with eto for 12 h. The boxed areas at the top are magnified at the bottom. The arrowheads indicate the ER-mitochondria contacts. ER endoplasmic reticulum; M mitochondrion. Scale bar, 500 nm. **d** Quantification of the ratio (%) of ER-mitochondria contacts (<30 nm) to the mitochondrial surface area and the average number of close contacts per mitochondrion in **c**. **e** U2OS cells stably expressing CFP-ER were transfected with either control (shControl) or EI24 shRNA (shEI24), treated with DMSO or eto for 12 h, probed with anti-TOM20 and anti-GFP antibodies, and subjected to PLA. Scale bar, 10 μm. **f** Quantification of the number of PLA-positive puncta/cell in **e**. **g** Representative EM images of wild-type (WT) and EI24-knockout (KO) U2OS cells treated with eto for 12 h. The boxed areas at the top are magnified at the bottom. Arrowheads indicate the ER-mitochondria contacts. ER, endoplasmic reticulum; M, mitochondrion. Scale bar, 500 nm. **h** Quantification of the ratio (%) of ER-mitochondria contacts (<30 nm) to the mitochondrial surface area and the average number of close contacts per mitochondrion in **g**. **i** Representative images of PLA results of U2OS cells stably expressing CFP-ER and transfected with either control or VDAC2 shRNA (shVDAC2), treated with DMSO or cpt for 12 h. Scale bar, 10 μm. **j** Quantification of the number of PLA-positive puncta/cell in **i**. **k** U2OS cells that stably expressed CFP-ER and overexpressed the vector, 3× Flag-EI24, 3× Flag-EI243m, 3× Flag-EI24ΔH1 or 3× Flag-EI24ΔH2 were subjected to PLA. Scale bar, 10 μm. **l** Quantification of the number of PLA-positive puncta/cell in **k**

We then tested the effects of p53 and DNA damage on ER-mitochondria contacts. p53 overexpression led to an increased number of PLA-positive puncta (Supplementary Information, Figure S8a and b). The number of PLA-positive puncta increased significantly after treatment with the DNA damaging drugs (Fig. 9a, b) whereas the protein levels of CFP-ER and TOM20 were not affected (Supplementary Information, Figure S8c). Electron microscopy further confirmed that the number and the closely apposed areas of ER-mitochondria contacts increased significantly upon DNA damage (Fig. 9c, d), thereby illustrating that DNA damage facilitates the formation of ER-mitochondria contacts.

EI24 knockdown reduced the number of ER-mitochondria contacts shown by PLA after treatment with the DNA damaging drug eto (Fig. 9e, f). Consistently, electron microscopic images showed that EI24 knockout reduced the number and the closely apposed area of ER-mitochondria contacts after eto treatment (Fig. 9g, h). Moreover, knockdown of VDAC2, but not VDAC1, decreased the number of PLA-positive puncta after exposure to DNA damaging drugs (Fig. 9i, j; Supplementary Information, Figure S8d-f). Notably, knockdown of neither VDAC2 nor EI24 affected the protein levels of CFP-ER or TOM20 (Supplementary Information, Figure S8g). Together, these results reveal that EI24 and VDAC2 are necessary for DNA damage-induced increase in ER-mitochondria contacts.

We next examined whether the hairpin region of EI24 is required for its ability to promote ER-mitochondria contacts. The mutant forms EI24-3m, EI24ΔH1 and EI24ΔH2 all showed impaired induction of ER-mitochondria contact formation in comparison to wild-type EI24 (Fig. 9k, l). The interactions between VDAC2 and these mutants were much weaker (Supplementary Information, Figure S8h). Therefore, the hairpin region of EI24 is required for it to promote ER-mitochondria contact formation.

### EI24 promotes DNA-damage induced apoptosis by facilitating ER-mitochondria Ca^2+^ transfer

**Fig. 10:**
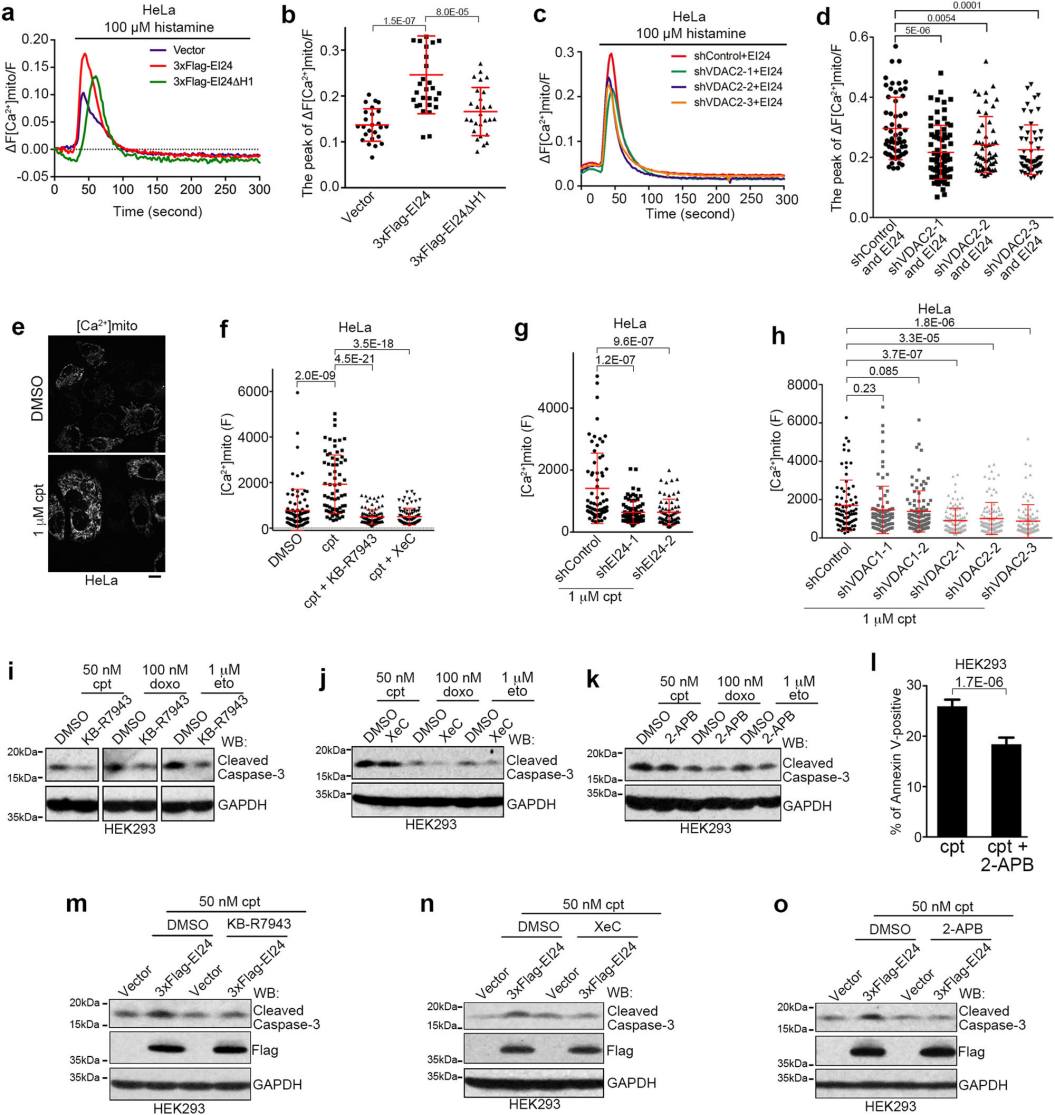
EI24 Promotes ER-mitochondria Ca^2+^ Transfer. **a** Measurement of the mito-R-GECO1.2 fluorescence intensity, which indicates mitochondrial Ca^2+^, after histamine stimulation in the control (Vector), 3× Flag-EI24 or 3× Flag-EI24ΔH1 overexpressing HeLa cells that stably expressed mito-R-GECO1.2. Traces show the mean ratio (ΔF/F) of the increased fluorescence intensity (ΔF) to the background fluorescence intensity (F) of the cells. Shown are the average traces from >20 cells in each group. **b** Quantification of the mito-R-GECO1.2 peak intensity in **a**. **c** Measurement of the mito-R-GECO1.2 fluorescence intensity, which indicates mitochondrial Ca^2+^, after histamine stimulation in stable mito-R-GECO1.2-expressing HeLa cells co-transfected with 3× Flag-EI24 and control (shControl) or VDAC2 shRNA (shVDAC2). **d** Quantification of the mito- R-GECO1.2 peak intensity in **c**. **e** Representative images of HeLa cells stably expressing mito-R-GECO1.2 and treated with DMSO or 1 μM cpt for 10 h. Scale bar, 10 μm. **f** Fluorescence intensity of HeLa cells stably expressing mito-R-GECO1.2 and treated with DMSO or 1 μM cpt, with or without 10 μM KB-R7943 or 1 μM XeC for 10 h. **g**, **h** Fluorescence intensity of HeLa cells stably expressing mito-R-GECO1.2, transfected with control, EI24 (shEI24, **g**), or VDAC1/2 shRNAs (**h**), and treated with DMSO or 1 μM cpt for 10 h. **i**–**k** Western blot analysis of HEK293 cells co-treated with 50 nM cpt, 100 nM doxo or 1 μM eto and and 10 μM KB-R7943 (**i**),1 μM Xec (**j**) or 5 μM 2-APB (**k**) for 12 h. GAPDH served as a loading control. **l** Quantification of flow cytometry analysis for cells positive for Annexin V. Cells were treated with 100 nM cpt and 10 μM KB-R7943 for 16 h. **m**–**o** Western blot analysis of HEK293 cells transfected with either the vector or 3× Flag-EI24 and treated with 50 nM cpt, and 10 μM KB-R7943 (**m**),1 μM Xec (**n**) or 5 μM 2-APB (**o**) for 12 h

ER-mitochondria contacts regulate mitochondrial division^49^ and mitochondrial morphology is closely related to apoptosis.^50^ However, EI24 overexpression did not change the fission rate of mitochondria (Supplementary Information, Figure S9a). Besides, levels of mitochondrial reactive oxygen species (ROS) is also closely related to apoptosis.^51^ EI24 knockout did not affect mitochondrial ROS levels either (Supplementary Information, Figure S9b). Finally, ER-mitochondria contacts are critical for Ca^2+^ transfer, and ER-mitochondria Ca^2+^ signaling is a key regulator in apoptosis.^16^ Therefore, we established HeLa cells that stably express cytosol-targeted GCaMP6s^52^ (cyto-GCaMP6s) and mitochondria-targeted R-GECO1.2^53^ (mito-R-GECO1.2) in which GCaMP6s or R-GECO1.2 acts as a sensor of Ca^2+^ concentration and emits the corresponding green or red fluorescence in the cytosol or mitochondria, respectively. We then used histamine to trigger Ca^2+^ release from the ER.^54^ EI24 overexpression did not affect cytosolic Ca^2+^ levels (Supplementary Information, Figure S9c and d) but increased mitochondrial Ca^2+^ uptake (Supplementary Information, Figure S9e-h). EI24 knockdown inhibited mitochondrial Ca^2+^ uptake, which was restored by re-introduction of shRNA-resistant EI24 (Supplementary Information, Figure S9i and j). Moreover, in contrast with wild-type EI24, overexpression of the EI24ΔH1 mutant resulted in a much weaker effect (Fig. 10a, b), suggesting that the hairpin region of EI24 is important for it to promote Ca^2+^ transfer from ER to mitochondria. VDAC2 knockdown also relieved the increase in mitochondrial Ca^2+^ uptake induced by EI24 overexpression (Fig. 10c, d), suggesting that EI24 promotes mitochondrial Ca^2+^ uptake by VDAC2.

To show whether EI24- and VDAC2-mediated ER-mitochondria contacts facilitate Ca^2+^ flux upon DNA damage, we tested the fluorescence intensity of mito-R-GECO1.2 after cpt treatment. Cpt treatment led to dramatically increased fluorescence intensity of mito-R-GECO1.2 (Fig. 10e), which was blocked by KB-R7943, an inhibitor of the inner mitochondrial membrane Ca^2+^ uniporter,^55,56^ or Xestospongin C (XeC), an inhibitor of the ER Ca^2+^ channel inositol-1,4,5-trisphosphate receptors (IP3Rs)^57^ (Fig. 10e, f). Knockdown of EI24 or VDAC2, but not VDAC1, significantly reduced the fluorescence intensity of mito-R-GECO1.2 after cpt treatment (Fig. 10g, h), suggesting that EI24 and VDAC2 are required for mitochondria Ca^2+^ uptake upon DNA damage. Furthermore, knockdown of VAPB, which interacts with PTPIP51 to form ER-mitochondria contacts,^58,59^ did not inhibit DNA damage-induced apoptosis or the increase in mitochondrial Ca^2+^ (Supplementary Information, Figure S9k and l). This suggests that the EI24-VDAC2 axis is independent of the previously reported VAPB-PTPIP51 connection.

We further investigated the relationship between calcium signaling and EI24-promoted apoptosis. Treatment with KB-R7943 or XeC inhibited the mitochondrial Ca^2+^ uptake promoted by EI24 overexpression (Supplementary Information, Figure S9e-h). Importantly, when mitochondrial Ca^2+^ uptake was impaired by KB-R7943, Xec or 2-APB, DNA damage-induced apoptosis was blocked (Fig. 10i–l; Supplementary Information, Figure S9m). Moreover, treatment with KB-R7943, Xec or 2-APB also blocked the effect of EI24 overexpression in promoting DNA damage-induced apoptosis (Fig. 10m–o), suggesting that the ER-mitochondria flux of Ca^2+^ is involved in this pathway.

Finally, we investigated whether EI24 interacts with IP3Rs. Immunoprecipitation showed that the transmembrane domains of both IP3RI and IP3RIII interacted with exogenous EI24 (Supplementary Information, Figure S10a-c). The transmembrane domains of IP3RIII co-immunoprecipitated with VDAC2 in the presence of wild-type EI24 but not EI24ΔC (Supplementary Information, Figure S10d). Moreover, endogenous VDAC2 was immunoprecipitated with endogenous IP3RIII at the presence of overexpressed EI24 (Supplementary Information, Figure S10e), suggesting that EI24 mediates the association between IP3RIII and VDAC2.

## Discussion

In this study, we show that DNA damage-activated p53 induces the up-regulation of REEP1/2 and EI24, which then promotes tubular ER formation, ER-mitochondria signal transduction, and subsequently apoptosis. Our results uncover striking morphological changes of ER upon DNA damage. A recent study reported that DNA damage triggers Golgi dispersal.^33^ Therefore, it is an intriguing question whether other organelles such as mitochondria, endosomes, and lysosomes also undergo morphological changes as an early response to DNA damage.

A previous report showed that a proportion of p53 localizes to ER-mitochondria contact sites and binds to the ER Ca^2+^ import channel SERCA2, thus regulating Ca^2+^ signaling during apoptosis, including DNA damage-induced apoptosis.^35^ Our data show that p53 indirectly regulates ER-mitochondria contacts and Ca^2+^ signaling by upregulating REEP1/2, EI24 and possibly VDAC2. Moreover, we also show that EI24 interacts with the transmembrane domain of ER Ca^2+^ export channel IP3Rs and promotes the association between IP3R and VDAC2. EI24 is also reported to interact with and regulate the function of SERCA2.^60^ Therefore, these two p53-mediated pro-apoptotic pathways may interconnect or promote each other. However, whether EI24 directly regulates p53 binding to SERCA2 or vice versa, or indeed, other precise aspects of the mechanism require further investigation.

It is an interesting question why only DNA damaging drugs, but not other apoptosis-inducing stimuli, such as TM, H_2_O_2_, or STS, induce tubular ER extension. Superficially, this can be simply explained by the fact that only DNA damage activates p53-mediated REEP1/2 and EI24 expression that facilitates tubular ER extension. However, it remains unclear why only the DNA damage pathway, but not other apoptosis pathways, employs tubular ER extension to promote apoptosis. One possible explanation would be that these apoptotic stimuli directly affect ER morphology in opposing directions. For example, TM and TG induce ER stress, which leads to an expansion of ER sheets.^61^ Another possibility is that DNA damage mainly affects nuclear material and, therefore, only DNA damage requires the p53-REEP1/2-EI24 pathway to affect organelle morphology, and this is one of the key players in cell survival and apoptosis.^62^ Nevertheless, a detailed interpretation of the evolutionary meaning of this p53-REEP1/2-EI24-tubular ER pathway awaits further investigation.

EI24 is a tumor suppressor protein^26,29^ and is often mutated in cancer cells.^24–27^ Loss of EI24 leads to resistance to DNA damage-induced cell death^29^ and dysfunction in DNA damage-induced apoptosis results in tumorigenesis and drug resistance in cancer cells.^63^ Combined with the observation that reduced Ca^2+^ transfer from the ER to mitochondria in cancer cells accelerates tumor growth,^64^ our findings may explain why EI24 is frequently lost or mutated in cancers. However, EI24 has been reported to promote cell growth and survival under normal conditions^65^ and in p53-deficient cells.^31^ We show that EI24 promotes apoptosis upon DNA damage, whereas it inhibited apoptosis after treatment with some other apoptotic drugs. In addition, EI24 has recently been reported to be crucial for autophagy and for promoting the degradation of some RING domain containing E3 ligases via the autophagy pathway,^66,67^ a process required for the elimination of misfolded proteins during ER stress.^68,69^ These findings provide a possible explanation for why EI24 appears to inhibit ER stress-induced apoptosis. Moreover, we show that knockdown of EI24 in untreated cells also affects the formation of ER-mitochondria contacts and Ca^2+^ flux. Mild increases of Ca^2+^ in mitochondria are able to stimulate the respiratory rate and are thus important for efficient synthesis of ATP in the cell.^70^ On the other hand, prolonged Ca^2+^increase leads to the opening of mitochondrial permeability transition pores and subsequent apoptosis.^16^ Therefore, it is possible that in normal cells, a low level of EI24 promotes mild ER-mitochondria Ca^2+^ signaling, thus promoting cell survival. In contrast, upon DNA damage, the expression of EI24 is significantly increased, triggering dramatic mitochondrial Ca^2+^ influx, and thus promoting apoptosis. Further investigation is required to determine the precise mechanism by which EI24 regulates apoptosis in response to different stimuli.

We show that upon DNA damage, EI24 is upregulated and interacts with VDAC2 to promote ER-mitochondria contacts, facilitating ER-mitochondria Ca^2+^ transfer and DNA damage-induced apoptosis. As the peripheral tubular ER is the major domain through which the ER contacts other organelles,^71^ it is possible that under DNA damage conditions, REEP1/2- and EI24-induced tubular ER extension allows EI24 to function as a MAM protein. Indeed, we observed that overexpression of EI24 in REEP1/2 knockdown cells promotes DNA damage-induced apoptosis whereas neither overexpression nor knockdown of REEP1/2 in EI24-knockout cells has any effect. Thus, EI24 is a key factor for DNA damage-induced apoptosis, while REEP1/2 are accessory elements that help to expand ER and facilitate EI24-mediated apoptosis. This may explain why overexpression of other ER shaping proteins, such as Rtn4, do not promote DNA-damage induced apoptosis and why only REEP1, but not REEP2, overexpression promotes DNA-damage induced apoptosis, since under normal conditions endogenous expression of REEP1 is hardly detectable, while REEP2 is much higher. Nevertheless, it is also possible that this may be due to REEP1-specific functions and indeed, REEP1 itself is reported to function in ER-mitochondria contacts.^15^

Our findings reveal that under DNA damage conditions, VDAC2, but not VDAC1, preferentially binds EI24 and is required for DNA damage-induced enhancement of ER-mitochondria contacts and apoptosis. Previous studies have shown that VDAC1 specifically regulates the ER-mitochondria flux of Ca^2+^ and apoptosis through the IP3RIII-GRP75-VDAC1 axis in response to ER and oxidative stress.^16^ Therefore, the formation and composition of ER-mitochondria contacts may be tightly regulated and highly relevant to physiological and pathological conditions.

We provide evidence that VDAC2 functions as a pro-apoptotic protein through ER-mitochondria Ca^2+^ transfer upon DNA damage. However, our results also show that VDAC2, like EI24, exerts an anti-apoptotic function after treatment with some other pro-apoptotic drugs, such as the ER stress-inducing drugs TM and TG, as well as the extrinsic apoptosis inducer TNFα. VDAC2 has been reported to bind to pro-apoptotic Bak and inhibit Bak-mediated apoptosis,^72^ but it is also required for tBid-induced apoptosis.^73^ Thus, VDAC2 may have a unique role in specific apoptotic pathways depending on its ability to interact with specific partners or on post-translational modifications under different physiological conditions. We also show that protein levels of VDAC2 are upregulated by p53 after DNA damage, although VDAC2 is not a direct transcriptional target of p53. Thus, there may be a strong connection between increased VDAC2 protein levels and the pro-apoptotic function of VDAC2 under DNA damage conditions. However, mechanisms underlying p53-mediated upregulation of the VDAC2 protein level in response to DNA damage requires further investigation.

## Materials and methods

### Cell culture and transfection

A431D, A549, BHK21, CHANG, CHO, COS7, H1299, HCT116, p53 KO HCT116, HEK293, HeLa, Hep3B, HepG2, Hey1B, Huh7, LO2, MCF7, MDA-MB-231, MEF, MHCC96-H, MHCC07-L, Mia PaCa 2, NRK, P19, PANC1, PK15, Ptk2, SH-Sy5y, SK-HEP1, SW13, U251, U2OS, and U87MG cells were cultured in DMEM (GIBCO) supplemented with 10% FBS (GIBCO or CellMax) at 37 °C in a humidified atmosphere of 5% CO_2_. AsPc1, BxPc3, C8161, SMMC7721, and SP20 cells were cultured in RPMI 1640 (GIBCO) supplemented with 10% FBS (GIBCO or CellMax) at 37 °C in a humidified atmosphere of 5% CO_2_. PEI was used to transiently transfect cells when they reached 50%-70% confluence. After transfection for 6 h, the medium was replaced with fresh medium.

### Reagents and antibodies

Camptothecin (cpt), doxorubicin (doxo), etoposide (eto), pifithrin-α (PFTα), and staurosporine (STS) were purchased from Selleckchem. Atto 647N-conjugated phalloidin, 2-aminoethoxydiphenyl borate (2-APB), EBSS, KB-R7943, puromycin (puro), thapsigargin (TG), TNFα, tunicamycin (TM), and Z-VAD-FMK were purchased from Sigma-Aldrich. G418 was purchased from GIBCO.

The primary antibodies used in this study were as follows: anti-actin (1: 100000, ABclonal, Cat. No. AC026), anti-atlastin3 (1:1000, Proteintech, Cat. No. 16921), anti-ATM (1:1000, Cell Signaling Technology, Cat. No. 2873), anti-ATR (1:1000, Proteintech, Cat. No. 19787), anti-bax (1:200, Santa Cruz Biotechnology, Cat. No. sc-493), anti-BiP (1:1000, Cell Signaling Technology, Cat. No. 3177), anti-calnexin (1:1000, Cell Signaling Technology, Cat. No. 2679), anti-calreticulon (1:1000, Proteintech, Cat. No. 10292), anti-calumenin (1:800, Proteintech, Cat. No. 17804), anti-CEPT1 (1:100, Proteintech, Cat. No. 20496), anti-cleaved caspase 3 (1:300, Cell Signaling Technology, Cat. No. 9664), anti-climp63 (1:5000 for western blot and 1:500 for immunofluorescence, Enzo, Cat. No. ALX-804-604), anti-β-COP (1:100, M3A5, a kind gift from Dr. Victor W. Hsu, Harvard University),^74^ anti-EI24 (1:1000, produced in rabbits using a peptide from EI24 (319–340: TSAEKFPSPHPSPAKLKATAGH) as an antigen), anti-fas (1:2000, Proteintech, Cat. No. 13098), anti-Flag (1:10000, Sigma-Aldrich, Cat. No. F1804), anti-GAPDH (1:5000, CWBIO, Cat. No. 0100A), anti-gadd45α (1:1000, Proteintech, Cat. No. 13747), anti-GFP (produced in-house, 1:10000),^75^ anti-giantin (1:10000, Abcam, Cat. No. ab24586), anti-HA (1:10000, Sigma-Aldrich, Cat. No. H9658), anti-Histone H2A.X (1:1000, Cell Signaling Technology, Cat. No. 7631), anti-IP3RIII (1:2000, BD Biosciences, Cat. No. 610312), anti-Kinectin (1:100 for IF, Proteintech, Cat. No. 19841), anti-Mfn2 (1:1000, Proteintech, 12186-1-AP), anti-MGMT (1:2000, Proteintech, Cat. No. 17195), anti-MOGAT2 (1:800, Proteintech, Cat. No. 19514), anti-phospho-ATM (S1981) (1:1000, Cell Signaling Technology, Cat. No. 5883), anti-phospho-ATR (S428) (1:1000, Cell Signaling Technology, Cat. No. 9718), anti-phospho-Histone H2A.X (S139) (1:1000, Cell Signaling Technology, Cat. No. 5883), anti-p21 (1:500, Ruiying Biological, Cat. No. RLM3453), anti-p27 (1:1000, Proteintech, Cat. No. 25614), anti-p53 (1:1000, Proteintech, Cat. No. 10442), anti-PARP (1:1000, Cell Signaling Technology, Cat. No. 9542), anti-paxillin (1:40000, BD Biosciences, Cat. No. 610052), anti-PDI (1:1000, Cell Signaling Technology, Cat. No. 3510), anti-PERK (1:500, Cell Signaling, 5683P), anti-REEP1 (1:3000, Proteintech, Cat. No. 17988), anti-REEP2 (1:3000, Proteintech, Cat. No. 15684), anti-REEP5 (1:1000, Proteintech, Cat. No. 14643), anti-Rtn4 (1:500 for western blot and 1:50 for immunofluorescence, Proteintech, Cat. No. 10740), anti-SERCA2 (1:1000, Cell Signaling Technology, Cat. No. 4388), anti-STIM1 (1:1000, Cell Signaling Technology, Cat. No. 4916), anti-TRAPα (1:500, Proteintech, Cat. No. 10583), anti-TOM20 (1:1000, BD Biosciences, 612278), anti-α-tubulin (1:5000, Sigma-Aldrich, Cat. No. T6199), anti-VAPB (1:1000, Proteintech, Cat. No. 14477), anti-VDAC1 (1:500, Proteintech, Cat. No. 10866), anti-VDAC2 (1:500, GeneTex, Cat. No. GTX114876), and anti-VDAC3 (1:500, Proteintech, Cat. No. 14451). Horseradish peroxidase-conjugated and Alexa Fluor 488/561-conjugated secondary antibodies (1:5000, Jackson ImmunoResearch) were used for western blot analysis and immunofluorescence experiments, respectively, according to the manufacturer instructions.

### Vector construction

The sequence coding human wild-type p53^76^ was a kind gift from Dr. Qi Ouyang (Center for Quantitative Biology, Peking University). p53 mutants were constructed by overlap PCR. VDAC2, REEP1 and REEP2 transcripts were obtained from the CCSB-Broad Lentiviral Expression Library hORFeome V8.1 (Thermo Fisher Scientific). Thereafter, shRNA-resistant REEP1/2 was obtained by overlap PCR, followed by cloning into the p3× Flag-CMV-14 vector (Sigma-Aldrich). For REEP1ΔH2, the second hairpin region of REEP1 (amino acids 43–65) was deleted by overlap PCR, followed by cloning into the pIRES2-EGFP vector (Clontech). HA-Rtn4a and Climp63-HA were obtained by cloning the corresponding transcripts into the pcDNA3.1 vector (Invitrogen) with a 5’-HA and 3’-HA tag, respectively. Climp63 was also cloned into the pCMV14 vector (Sigma-Aldrich) to generate Climp63-3× Flag. The sequences encoding the human EI24 and Lnp1 transcripts were amplified from HEK293 cell cDNA and cloned into the p3× Flag-CMV-7.1 (Sigma), pEGFP-C2 (Clontech), or pEGFP-N3 (Clontech) vectors. EI24 mutants were then constructed using overlap PCR. The sequences encoding the transmembrane domains of human IP3RI and IP3RIII were amplified from HEK293 cell cDNA and cloned into the pEGFP-C2 (Clontech) vector. The sequences encoding the transmembrane domains of human IP3RI and IP3RIII were amplified from HEK293 cell cDNA and cloned into the pEGFP-C2 (Clontech) vector.

The sequences encoding GCaMP6s^52^ and R-GECO1.2^53^ were kind gifts from Yulong Li (College of Life Sciences, Peking University). To construct mito-R-GECO1.2, R-GECO1.2 was constructed by adding the N-terminal mitochondrial transit peptide of cytochrome *c* oxidase subunit 8A (amino acids 1–29: MSVLTPLLLRGLTGSARRLPVPRAKIHSL) at the N-terminus of R-GECO1.2 followed by cloning into the pcDNA3.1 (Invitrogen) vector. To construct cyto-GCaMP6s, GCaMP6s was cloned into the pcDNA3.1 (Invitrogen) vector. The ER marker mCherry-ER was obtained by adding the signal peptide of calumenin^75^ at the N-terminus and the “KDEL” ER retrieval signal at the C-terminus of mCherry, followed by cloning into the pcDNA3.1 vector. CFP-ER was obtained by ligation of CFP with the C-terminal ER localization sequence of yeast Ubc6 (amino acids 233–250: MVYIGIAIFLFVGLFMK) as reported^48^ and constructed into the pcDNA3.1 vector. Mito-GFP (mtGFP) was obtained by fusing GFP to the mitochondrial targeting sequence derived from the precursor of subunit VIII of human cytochrome C oxidase (from pDsRed2-Mito Vector, Clontech 632421), Mito-BFP (mtBFP) was obtained by adding four mitochondrial targeting sequences of the precursor of subunit VIII of human cytochrome C oxidase to the N-terminal of two consecutive mTagBFP2 (gift from Dr. Yulong Li at the College of Life Sciences of Peking University).

### Fluorescent microscopy

Cells were grown to 50% confluence on a coverslip, fixed in 4% paraformaldehyde for 20 min at 37 °C, and permeabilized with 0.15% Triton X-100 for 10 min. The cells were then blocked with 3% bovine serum for 30 min, incubated with primary antibodies overnight at 4 °C, and then stained with secondary antibodies for 1 h at room temperature. Samples were observed under a Zeiss LSM-710NLO confocal fluorescent microscope equipped with a 100 × /1.42 NA oil immersion objective lens, a Zeiss LSM 880 Laser Scanning Microscope equipped with a 63 × /1.40 NA oil immersion objective lens using the Airyscan function, or a GE Healthcare DeltaVision OMX SR imaging system (GE) equipped with a 63 × /1.40 NA oil immersion objective lens using 3D-SIM function.

For time lapse observation, cells were transfected with mCherry-KDEL, treated with 50 μM eto 8 h after transfection, and observed under a spinning disc confocal microscope (PerkinElmer) equipped with a 100 × /1.40 NA oil immersion objective lens. Images were captured at 5-min intervals. Noise was removed from the captured images using Volocity software (PerkinElmer).

### Analysis of ER morphology

For the analysis of the ER distribution area, cells were transfected with the ER marker mCherry-ER and treated with DMSO, eto, cpt or doxo 8 h after transfection. 16 h after drug treatment, the cells were observed under an IX71 fluorescent microscope equipped with a 20 × /0.45 NA immersion objective lens (Olympus) and images were captured using a DP controller and DP manager software (Olympus). The ER distribution area was then marked and calculated using ImageJ software (NIH).

For analysis of the detailed ER morphology, COS7 or U2OS cells transfected with mCherry-ER and having had the indicated treatments were fixed and observed under a Zeiss LSM-710NLO confocal fluorescent microscope equipped with a 100 × /1.42 NA oil immersion objective lens. Images were captured with ZEN9 software (Zeiss). For analyzing the density of three-way-junctions, a 200 μm^2^ area with the sparsest tubular ER network of each cell was chosen, and the number of three-way junctions inside this area were counted.

The distribution areas of the nucleus, peripheral and perinuclear ER were outlined and calculated using ImageJ software as indicated in the Supplementary Information, Figure S1c. To specifically distinguish between the peripheral and perinuclear ER, the threshold function within ImageJ was used as previously described.^39^

For analysis of the volume of Rtn4 and Climp63-positive ER, cells were fixed 16 h after drug treatment and co-immunolabeled with anti-Rtn4 and anti-climp63 antibodies, followed by labeling with fluorescent probe-conjugated secondary antibodies. Samples were then observed under a spinning disc confocal microscope (PerkinElmer) equipped with a 100 × /1.40 NA oil immersion objective lens. Images were captured using Volocity software (PerkinElmer). Images were captured for multiple layers starting at the cell base and ending at the cell apex at 0.1-μm intervals. Captured images were then cropped to show single cells, followed by deconvolution, threshold determination, 3D reconstruction, and volume calculation using Volocity software (PerkinElmer). For the analysis of total ER volume and cell volume, U2OS cells were transfected with mCherry-ER and GFP (indicating the whole cell), treated with eto or cpt for 16 h, and then fixed for observation and 3D reconstruction using a spinning disc confocal microscope or captured by the DeltaVision OMX SR imaging system (GE) using 3D-SIM function and analyzed by Volocity software.

### RNAi

The shRNA sequences in the pLKO.1 vector used to target each protein were as follows: EI24 (5′-ggttcaataaaggaattga-3′ and 5′-gtggtcttcttaagcaaca-3′, designed by GenePharma), p53 (5′-gactccagtggtaatctac-3′ and 5′-taccaccatccactacaacta-3′),^77,78^ REEP1 (5′-gacatcttcctttgttggttt-3′ and 5′-agtttgtacatcccacactat-3′, designed by Sigma-Aldrich), and REEP2 (5′-caagagctatgagaccatgat-3′ and 5′-actggcttccaagacactgaa-3′, designed by Sigma-Aldrich), Rtn4 (5′-gggcatatctggaatctga-3′, 5′-ccatcagctttaggatata-3′ and 5′-gggtgtgatccaagctatc-3′),^79^ VDAC2 (5′-aaggatgatctcaacaagagc-3′, 5′-gcagctaaatatcagttggat-3′ and 5′-caaggtttgaaactgacattt-3′),^17,80^ and VDAC1 (5′-aagcgggagcacattaacctg-3′, 5′-ctccaggttaaagttgattca-3′).^17,81^ The siRNAs (5′-gcucuuggcucuggugguu-3′ and 5′-uguuacagccuuucgauua-3′) against VAPB as reported^58,59^ were also used. For rescue assays, cells were transfected with corresponding shRNAs. 48 h after the first transfection, the cells were co-transfected with shRNAs and shRNA-resistant plasmids and then subjected to further drug treatment and analysis.

U2OS cells with REEP1/2 stable knockdown were prepared by transfecting with corresponding shRNAs and treating with 2 μg/mL puromycin 24 h after transfection. Puromycin treatment continued for approximately two weeks until no further cells died. Lysates from the surviving cells were then tested for efficient knockdown efficiency by western blot analysis.

The shRNA-resistant form of EI24 was obtained using overlap PCR, and the construct was cloned into the p3× Flag-CMV-14 vector (Sigma). The shRNA-targeted regions of EI24 were mutated into 5′-ggtttaacaagggtatcga-3′ and 5′-gtcgtatttctaagtaata-3′. For the rescue assays, the cells were transfected with shRNAs, co-transfected with shRNAs and shRNA-resistant plasmids 48 h after the first transfection and subjected to further drug treatment and analysis.

### Genome-editing of REEP1/2 and EI24

For generating REEP1/2 double knockout U2OS cells, target oligos (5′-cctggagtacttaccaacaa-3′ and 5′-gtctgtgaatgtctctgctg-3′ for REEP1, and 5′-gccaaagatgagcctaggg-3′ and 5′-ggaagaataggctgggtac-3′ for REEP2) were synthesized and ligated into the gRNA vector.^82^ The gRNA vectors were then transfected into U2OS cells together with Cas9^82^ and pEGFP-C2 plasmids. Four days later, GFP-positive cells were selected and cloned by flow cytometry (BeckMan Coulter, MoFlo XDP). Following proliferation, the clones were subjected to western blot analysis to confirm successful knockout.

To establish EI24-knockout U2OS cells, the target oligos (5′-gagaggagcagcgtcgaaga-3′ and 5′-gaagagcccagagtatagag-3′) were synthesized (Thermo Fisher) and ligated into the gRNA vector.^82^ The gRNA vectors were then transfected into U2OS cells together with Cas9^82^ and the pEGFP-C2 plasmids. Four days later, GFP-positive cells were selected and cloned using flow cytometry (Beckman Coulter, MoFlo XDP). The expanded clones were then subjected to genomic DNA extraction, PCR amplification, and sequencing.

To generate EI24-mutant U2OS cells, the target oligo 5′-TAACCAGCTGACCGACAAGA-3′ was used. The mutation template was cloned from U2OS genomic extract and mutated using overlap PCR. The gRNA, Cas9, and template plasmids were transfected into U2OS cells together with pEGFP-C2, and EGFP-positive cells were selected four days later. Successful mutations were selected after proliferated subclones were sequenced.

### In vivo experiments

All animal experiments were undertaken in accordance with the National Institute of Health Guide for the Care and Use of Laboratory Animals, and with the approval of the Committee of Animal Research in Peking University. Animals were kept in a specific pathogen-free environment in the Peking University Laboratory Animal Center. p53 knockout and corresponding wild-type mice (6 weeks old male, C57BL/6) were obtained from Vitalstar. Doxo (3 mM, 200 µL) was intraperitoneally injected into wild-type or p53 knockout mice, which were sacrificed 16 h after injection. A piece of liver from a similar location from each mouse was used for western blot analysis.

### Electron microscopy

Cells were cultured as described above. U2OS cells were fixed using 2% paraformaldehyde and 2% glutaraldehyde in PBS buffer (pH 7.4) overnight at 4 °C. All samples were washed three times with PBS buffer (pH 7.4) and post-fixed in 1% osmium tetroxide and 1.5% potassium ferrocyanide on ice for 30 min in the same buffer. The cells were then washed three times with distilled water and stained with 1% uranyl acetate in 50% ethanol at room temperature for 1 h. The samples were dehydrated in a graded ethanol series (65%, 75%, 85%, 95%, 99%, and 100%) and embedded into Epon 812 resin (SPI-Chem). Sections were cut on a Leica UC7 Ultramicrotome and viewed with a transmission electron microscope (JEOL, JEM 1010).

### Subcellular fractionation

The procedure used for subcellular fractionation was conducted as described in a previous report.^83^ Briefly, 100 plates (10-cm diameter) containing HEK293T cells were collected in ice-cold IB_cells_-1 buffer (225 mM mannitol, 75 mM sucrose, 0.1 mM EGTA, and 30 mM Tris-HCl, pH 7.4) and gently disrupted using a Dounce homogenizer (approximately 100 times). The homogenate was centrifuged twice at 600× *g* for 5 min at 4 °C, and the supernatant was centrifuged at 7000×* g* for 10 min at 4°C to pellet the crude mitochondrial extraction. The resultant supernatant was further centrifuged at 100,000× *g* for 1 h at 4°C (Beckman 70-Ti rotor), which separated the ER (pellet) and cytosolic fractions (supernatant). The crude mitochondrial pellet was gently resuspended in 2 mL of ice-cold MRB buffer (250 mM mannitol, 5 mM HEPES and 0.5 mM EGTA, pH 7.4), layered on 8 mL of Percoll medium (225 mM mannitol, 25 mM HEPES, 1 mM EGTA and 30% Percoll (v/v), pH 7.4) in a 14 mL thin-wall Polyallomer ultracentrifuge tube and centrifuged at 95,000× *g* for 30 min at 4°C (Beckman SW40 rotor) to separate the mitochondria-associated ER membrane (MAM) and pure mitochondria. The quality of the subcellular fractionation was evaluated by western blot using markers corresponding to the components obtained in the fractionation.

### Proximity ligation assay (PLA)

For the analysis of ER-mitochondria contacts, CFP with a C-terminal transmembrane domain of Ubc6^84^ (CFP-ER) was used to mark the ER so that CFP faced the cytosol, and the mitochondrial outer membrane protein TOM20 was used to mark the mitochondria. U2OS cells were transfected with CFP-ER and other corresponding plasmids, and subjected to the PLA assay (Sigma-Aldrich, Cat. No. DUO92008) according to the manufacturer’s instructions with anti-GFP and anti-TOM20 antibodies. Samples were observed under an IX71 fluorescent microscope equipped with a 60 × /1.42 NA oil immersion objective lens, and the number of PLA positive dots was quantified using the particle analysis function of ImageJ software (National Institutes of Health) and expressed as dots per cell.

### Ca^2+^ imaging

HeLa cells stably expressing mitochondria-targeted R-GECO1.2 (mito-R-GECO1.2), which acts as mitochondria Ca^2+^ sensor and emits red fluorescence, or cyto-GCaMP6s, which acts as cytosol Ca^2+^ sensor and emits green fluorescence, were used for the Ca^2+^ imaging experiments. Ca^2+^ imaging was performed with a spinning-disk UltraVIEW VoX imaging system (Perkin Elmer) equipped with a 20 × /0.75 NA objective lens (Nikon).

For histamine-induced Ca^2+^ dynamics, cells were washed twice with Hank’s balanced salt solution, followed by the addition of Ca^2+^ buffer (150 mM NaCl, 5.4 mM KCl, 20 mM HEPES, 10 mM glucose, 1 mM MgSO_4_, and 1.8 mM CaCl_2_, pH 7.4), immediately before imaging. Cells were excited at either 561 nm (for mitochondria Ca^2+^) or 488 nm (for cytosol Ca^2+^), and images were acquired every 5 s for 5 min. Approximately 30 s after the start of the experiment, histamine was added at a final concentration of 100 μM. Images were post-processed with Volocity (Perkin Elmer).

For mitochondria Ca^2+^ upon DNA damage, cells were treated with 1 μM cpt for 10 h before imaging. Images were acquired at 30 points randomly, and post-processed and analyzed with Volocity (Perkin Elmer).

### Immunoprecipitation and western blot

For the immunoprecipitation analysis, HEK293T cells were transfected with the appropriate plasmids. Cells were collected and lysed in immunoprecipitation buffer (50 mM Tris, 150 mM NaCl, 1% NP-40, 1 mM DTT, and 5 mM EDTA, pH 8.0). After centrifugation at 12,000×* g* for 10 min at 4 °C, the lysates were incubated with the appropriate antibody overnight at 4 °C and then incubated with Protein G or A Sepharose (Amersham) for 2 h. The immunoprecipitated samples were then analyzed by western blot.

For the western blot assays, the proteins were separated by SDS-PAGE and transferred onto polyvinylidene difluoride membranes (Millipore). The membranes were blocked, probed with primary and secondary antibodies, and exposed using a film processing machine (Kodak) or an Amersham Imager 600 chemiluminescence imager (GE Healthcare). The band intensity was analyzed by ImageJ (NIH) as previously reported.^75^

### Flow cytometry

Cell cycle analysis was performed as previously described.^85^ In brief, transfected U2OS cells were treated with 5 μM eto for 16 h, fixed with 70% alcohol for 2 h, treated with 0.1 mg/mL RNase for 20 min, re-suspended in 50 μg/mL propidium podide, and analyzed by flow cytometry (BeckMan Coulter, MoFlo XDP).

For apoptosis analysis, cells were harvested and washed in PBS, re-suspended in 1× binding buffer (10 mM HEPES, 150 mM NaCl, 2.5 mM CaCl2, pH 7.4) containing Annexin V (Invitrogen A13201), and incubated in dark for 30 min according to the manufacturer’s instructions. Then cells were loaded with propidium iodide (PI) and analyzed by flow cytometry with the BD FACSVerse system. The results were analyzed by FlowJo software.

### Microtubule sedimentation assay

To analyze the polymerization status of microtubules, cells were treated with 1 μM eto for 16 h, harvested, and then lysed in buffer (25 mM HEPES, 150 mM KAc, 2 mM Mg(Ac)_2_, 0.5% Triton X-100, pH 7.4) at 37 °C. The cell lysates were then centrifuged at 100,000×* g* for 30 min. The resulting supernatants and pellets were analyzed by western blot.

### MTT assay

Cells were seeded in 96-well plates, transfected and treated as indicated, and assayed using the CellTiter 96 AQueous One Solution Cell Proliferation Assay (Promega, Cat. No. G3581) according to the manufacturer instructions. To exclude the influence of nutrient deficiency or drug degradation, the culture medium containing drugs was changed daily. Each experiment was repeated 8 times.

### RNA isolation, RT-PCR, and qRT-PCR

Total RNA was extracted with TransZol reagent (TransGen, Cat. No. ET111-01) as previously described.^86^ Thereafter, total RNA was reverse transcribed using the GoldScipt cDNA Synthesis Kit (Invitrogen, Cat. No. C81401190) and subjected to quantitative RT-PCR analysis in an ABI 7300 Detection System (Applied Biosystems) using the SYBR Green PCR Master Mix (Applied Biosystems, Cat. No. 4344463) according to the manufacturer’s instructions. GAPDH served as reference, and the 2^−ΔΔCT^ method^87^ was used to analyze the data. The primers used for qRT-PCR were as listed in Supplementary Information, Table S1.

### Luciferase assay

For the luciferase assay, DNA fragments containing the REEP1/2 promoter region were cloned into the pGL3-basic reporter vector (Promega) expressing firefly luciferase. The cells were transfected with reporter vectors and renilla luciferase expressing internal control vectors. At 30 h after transfection, luciferase activity was analyzed by the Dual-Luciferase Reporter Assay System (Promega, Cat. No. E1910) according to the manufacturer’s instructions. In brief, the cells were lysed in passive lysis buffer for 1 h, and the cell lysates were transferred to a 96-well culture plate for the measurement of luciferase activity. The relative luciferase level was calculated as reporter activity.

### Chromosome immunoprecipitation

Chromosome immunoprecipitation was performed as previously described.^88^ In brief, cells were cross-linked with 1% formaldehyde and lysed, and the nuclear fraction was further sonicated and centrifuged. The supernatant containing chromatin was then incubated with an antibody, followed by the addition of Protein A Sepharose beads (GE Healthcare, Cat. No. 17-0963-03). After extensive washing, the immunoprecipitated complex was eluted, reverse cross-linked, and subjected to DNA extraction with a Gel Extraction Kit (CWBIO, Cat. No. CW 2302A). The purified DNA fraction was then analyzed by PCR using specific primers.

### Reactive oxygen species (ROS)

For ROS analysis, wild-type and EI24 knockout cells were first collected and washed with PBS to remove traces of the original medium, followed by an incubation in serum-free medium containing 2 μM H2DCFDA (Invitrogen, D399) for 30 min in 37 °C with 5% CO_2_. Cells were then trypsinized and resuspended in PBS, with or without 50 μM H_2_O_2_ for 30 min on ice and subjected to flow cytometry analysis on the BD FACSVerse system. The results were analyzed by FlowJo software.

### Statistics and reproducibility

No statistical method was used to predetermine sample size. The experiments were not randomized. Data were analyzed in a double-blinded manner. Statistical data were analyzed using unpaired two-tailed Student’s *t*-tests. Data are represented as means ± S.D. with *p* value shown on top. When representative images are shown, at least three repeats were performed.

### Data availability

All data supporting the findings of this study are available from the corresponding author on request.

## Electronic supplementary material

Supplementary information, Vedio S1

Supplementary information, Vedio S2

Supplementary video legend

Supplementary information, Figure S1

Supplementary information, Figure S2

Supplementary information, Figure S3

Supplementary information, Figure S4

Supplementary information, Figure S5

Supplementary information, Figure S6

Supplementary information, Figure S7

Supplementary information, Figure S8

Supplementary information, Figure S9

Supplementary information, Figure S10

Supplementary Information, Table S1
