# Supplementary material for: DNA damage triggers tubular endoplasmic reticulum extension to promote apoptosis by facilitating ER-mitochondria signaling
Source: Cell Res. 2018 Jul 20;28(8):833–54. doi: 10.1038/s41422-018-0065-z (PMC6063967; doi:10.1038/s41422-018-0065-z)
Supplement: Supplementary file 3 — Supplementary video legend [file 41422_2018_65_MOESM3_ESM.pdf]

Videos S1 and S2. DNA Damage Triggers Tubular ER Extension, Related to Figure 1. Representative time-lapse video recording of COS7 cells transfected with mCherry-ER and treated with 50 μM eto. The indicated times are shown as hour: minute. Scale bar, 10 μm.
