# Supplementary material for: DNA damage triggers tubular endoplasmic reticulum extension to promote apoptosis by facilitating ER-mitochondria signaling
Source: Cell Res. 2018 Jul 20;28(8):833–54. doi: 10.1038/s41422-018-0065-z (PMC6063967; doi:10.1038/s41422-018-0065-z)
Supplement: Supplementary file 4 — Supplementary information, Figure S1 [file 41422_2018_65_MOESM4_ESM.pdf]

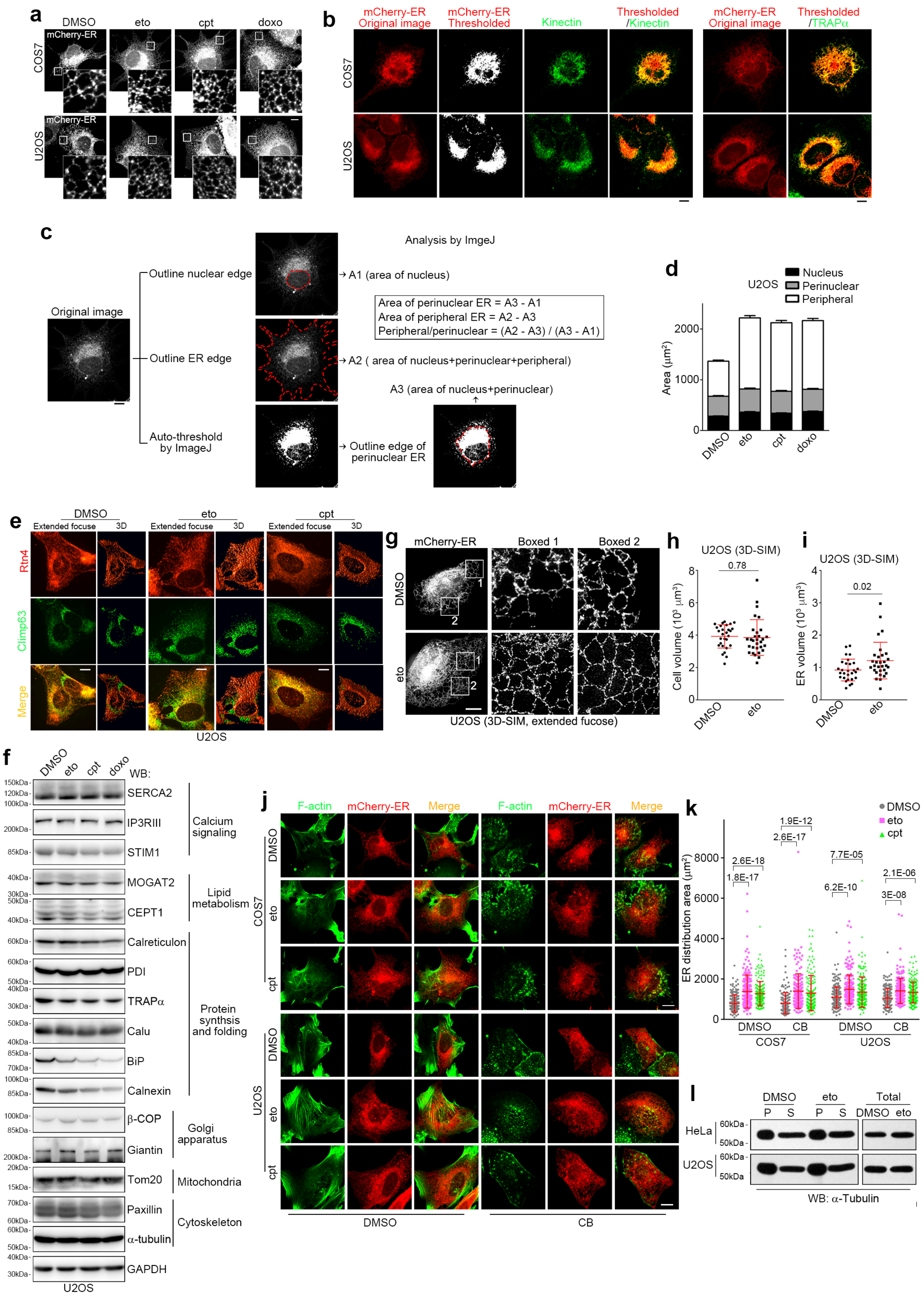

a
DMSO
eto
cpt
doxo
COS7
U2OS
mCherry-ER
b
mCherry-ER Original image
mCherry-ER Thresholded
Kinectin
Thresholded /Kinectin
mCherry-ER Original image
Thresholded /TRAPα
c
Analysis by ImgeJ
Original image
Outline nuclear edge
A1 (area of nucleus)
Area of perinuclear ER = A3 - A1
Area of peripheral ER = A2 - A3
Peripheral/perinuclear = (A2 - A3) / (A3 - A1)
Outline ER edge
A2 ( area of nucleus+perinuclear+peripheral)
A3 (area of nucleus+perinuclear)
Auto-threshold by ImageJ
Outline edge of perinuclear ER
d
U2OS
Nucleus
Perinuclear
Peripheral
Area (μm²)
e
Extended focuse
3D
Rtn4
Climp63
Merge
U2OS
g
mCherry-ER
Boxed 1
Boxed 2
U2OS (3D-SIM, extended fucose)
h
U2OS (3D-SIM)
Cell volume (10³ μm³)
0.78
i
U2OS (3D-SIM)
ER volume (10³ μm³)
0.02
f
WB:
SERCA2
IP3RIII
STIM1
Calcium signaling
MOGAT2
CEPT1
Lipid metabolism
Calreticulon
PDI
TRAPα
Calu
BiP
Calnexin
Protein synthesis and folding
β-COP
Giantin
Golgi apparatus
Tom20
Mitochondria
Paxillin
α-tubulin
Cytoskeleton
GAPDH
U2OS
j
F-actin
mCherry-ER
Merge
CB
k
ER distribution area (μm²)
1.8E-17
2.6E-18
2.6E-17
1.9E-12
6.2E-10
7.7E-05
3E-08
2.1E-06
COS7
l
P
S
Total
HeLa
WB: α-Tubulin

**Figure S1. DNA Damage Triggers Tubular ER Extension and Methodology for ER Morphology Analysis.**
(a) Representative images of the ER in COS7 and U2OS cells treated with DMSO, 50 μM eto, 1 μM cpt, or 1 μM doxo for 16 hours. Scale bar, 10 μm. The outlined area is magnified at the lower right of each panel.
(b) Representative images of U2OS and COS7 cells transfected with mCherry-ER, and immuno-stained with anti-Kinectin or anti-TRAPα antibodies. Scale bars, 10 μm.
(c) Schematic figure illustrating the analysis of the distribution area for ER structures.
(d) Areas of different ER structures in U2OS cells treated with DMSO, 50 μM eto, 1 μM cpt or 1 μM doxo for 16 hours. $n \geq 120$ cells.
(e) Representative images of extended focus (left panel) or 3D reconstruction (right panel) results of fluorescently co-immunolabeled Rtn4 (red) and Climp63 (green) in U2OS cells treated with DMSO, 50 μM eto or 1 μM cpt for 16 hours. Data are captured by spinning disc microscopy and deconvolved by Volocity. Scale bars, 10 μm.
(f) Western blot analysis of lysates from U2OS cells treated with DMSO, 50 μM eto, 1 μM cpt or 1 μM doxo for 16 hours. GAPDH served as a loading control. SERCA2, sarco/endoplasmic reticulum $Ca^{2+}$-ATPase; IP3RIII, inositol 1,4,5-trisphosphate (IP3) receptor III; STIM1, stromal interaction molecule 1; MOGAT2, monoacylglycerol *O*-acyltransferase 2; CEPT1, choline/ethanolamine phosphotransferase 1; PDI, protein disulfide isomerase; TRAPα, translocon-associated protein α; BiP, Ig binding protein.
(g) Representative 3D-SIM images of mCherry-ER in U2OS cells treated with DMSO or 50 μM eto for 16 hours. Images are shown in the extended focus manner. Boxed regions are magnified. Scale bar, 10 μm.
(h, i) Statistical analyses of the volumes of total cell (h) and ER (i) from 3D-SIM images of GFP and mCherry-ER in U2OS cells treated with DMSO or 50 μM eto for 16 hours. $n \geq 27$ cells.
(j) COS7 (upper panel) or U2OS (lower panels) cells transfected with mCherry-ER were treated with DMSO, 50 μM eto or 1 μM cpt in the absence or presence of 5 μM cytochalasin B (CB) for 16 hours, and labeled with phalloidin (green) to show actin filaments (F-actin). Scale bar, 10 μm.
(k) Corresponding analyses of the ER distribution area shown in (j).
(l) Assays to determine the status of microtubule polymerization in HeLa and U2OS cells treated with 50 μM eto for 16 hours. After centrifugation, pellet (P) and supernatant (S) fractions were subjected to western blot (WB) analysis.
