# Supplementary material for: DNA damage triggers tubular endoplasmic reticulum extension to promote apoptosis by facilitating ER-mitochondria signaling
Source: Cell Res. 2018 Jul 20;28(8):833–54. doi: 10.1038/s41422-018-0065-z (PMC6063967; doi:10.1038/s41422-018-0065-z)
Supplement: Supplementary file 5 — Supplementary information, Figure S2 [file 41422_2018_65_MOESM5_ESM.pdf]

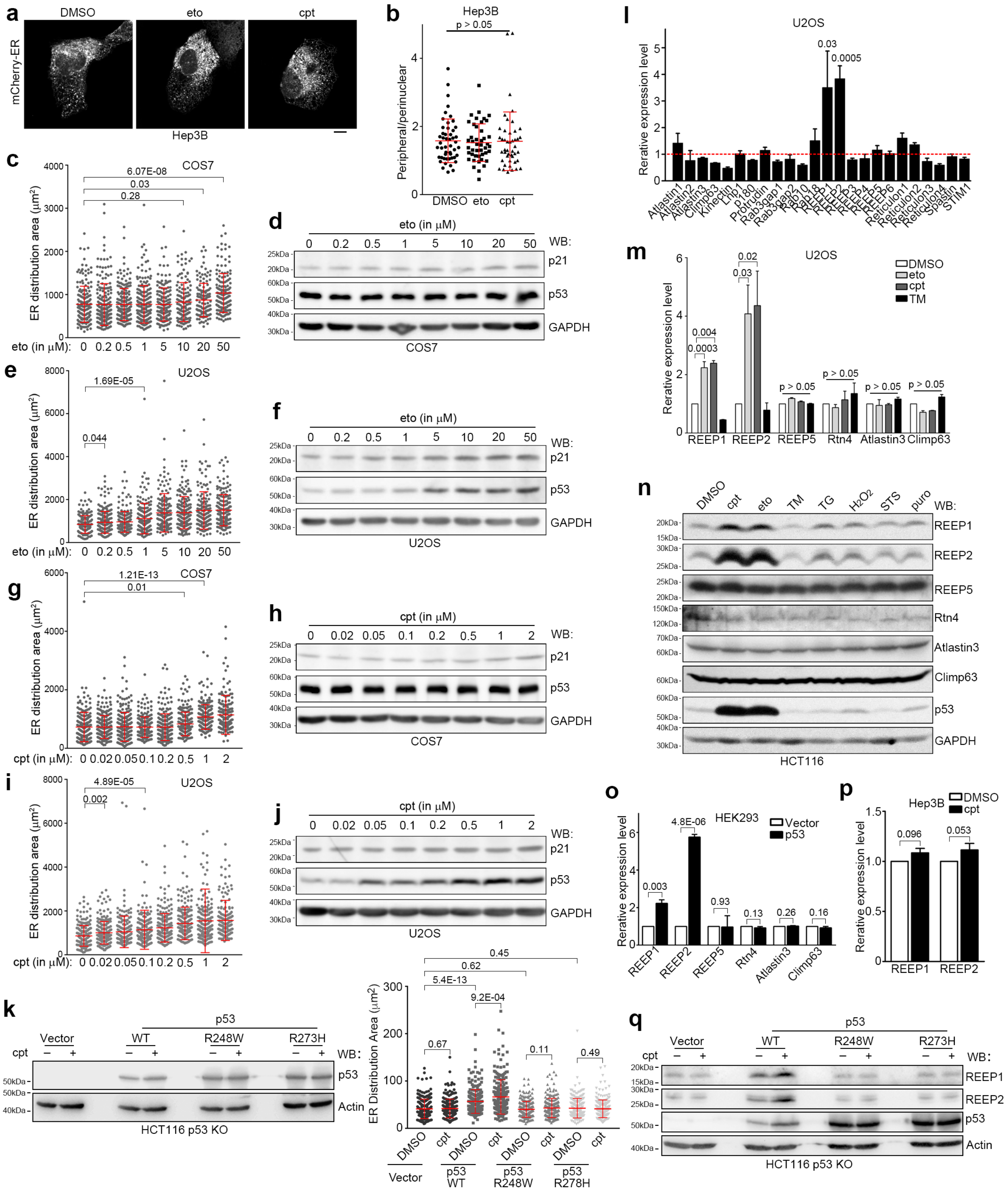

Figure S2. DNA Damage Promotes Tubular ER Extension via p53, and REEP1/2 are Downstream Targets of p53.

(a, b) Representative images (a) and statistical analysis of the distribution ratios of peripheral/perinuclear ER (b) in Hep3B cells treated with DMSO, 50 μM eto or 1 μM cpt for 16 hours. Scale bar, 10 μm. n ≥ 50 cells.

(c-j) ER distribution areas (total area of mCherry-ER) and western blot (WB) analyses of lysates from COS7 or U2OS cells exposed to the indicated doses of eto or cpt. $n \geq 200$ cells. GAPDH served as the loading control.

(k) Corresponding western blot analysis (left) and ER distribution area (right) in HCT116 p53 knockout (KO) cells overexpressing p53 or its mutants and treated with DMSO or 100 nM cpt for 16 hours.

(l) Quantitative real-time PCR analysis of the mRNA level of proteins involved in ER-shaping in U2OS cells exposed to 1 μM cpt for 16 hours. $n = 3$.

(m) Quantitative real-time PCR analysis of the mRNA levels for REEP1/2/5, Rtn4, atlastin3, and Climp63 in U2OS cells exposed to DMSO, 50 μM eto, 1 μM cpt or 2 μM TM for 16 hours. $n = 3$.

(n) Western blot analysis of lysates from HCT116 cells treated with DMSO, 100 nM cpt, 1 μM eto, 1 μM TM, 100 nM TG, 0.5 mM H_2O_2, 50 nM STS or 500 ng/mL puromycin (puro) for 16 hours.

(o) HEK293 cells overexpressing control (vector) or p53 were analyzed for the mRNA levels of the indicated proteins by quantitative real-time PCR. $n = 3$.

(p) Hep3B cells were treated with DMSO or 1 μM cpt for 16 hours, and the REEP1/2 mRNA level was analyzed by quantitative real-time PCR. $n = 3$.

(q) Western blot analysis of lysates from HCT116 p53 knockout cells transfected with p53 or its mutants and treated with DMSO or 100 nM cpt for 16 hours.
