# Supplementary material for: DNA damage triggers tubular endoplasmic reticulum extension to promote apoptosis by facilitating ER-mitochondria signaling
Source: Cell Res. 2018 Jul 20;28(8):833–54. doi: 10.1038/s41422-018-0065-z (PMC6063967; doi:10.1038/s41422-018-0065-z)
Supplement: Supplementary file 6 — Supplementary information, Figure S3 [file 41422_2018_65_MOESM6_ESM.pdf]

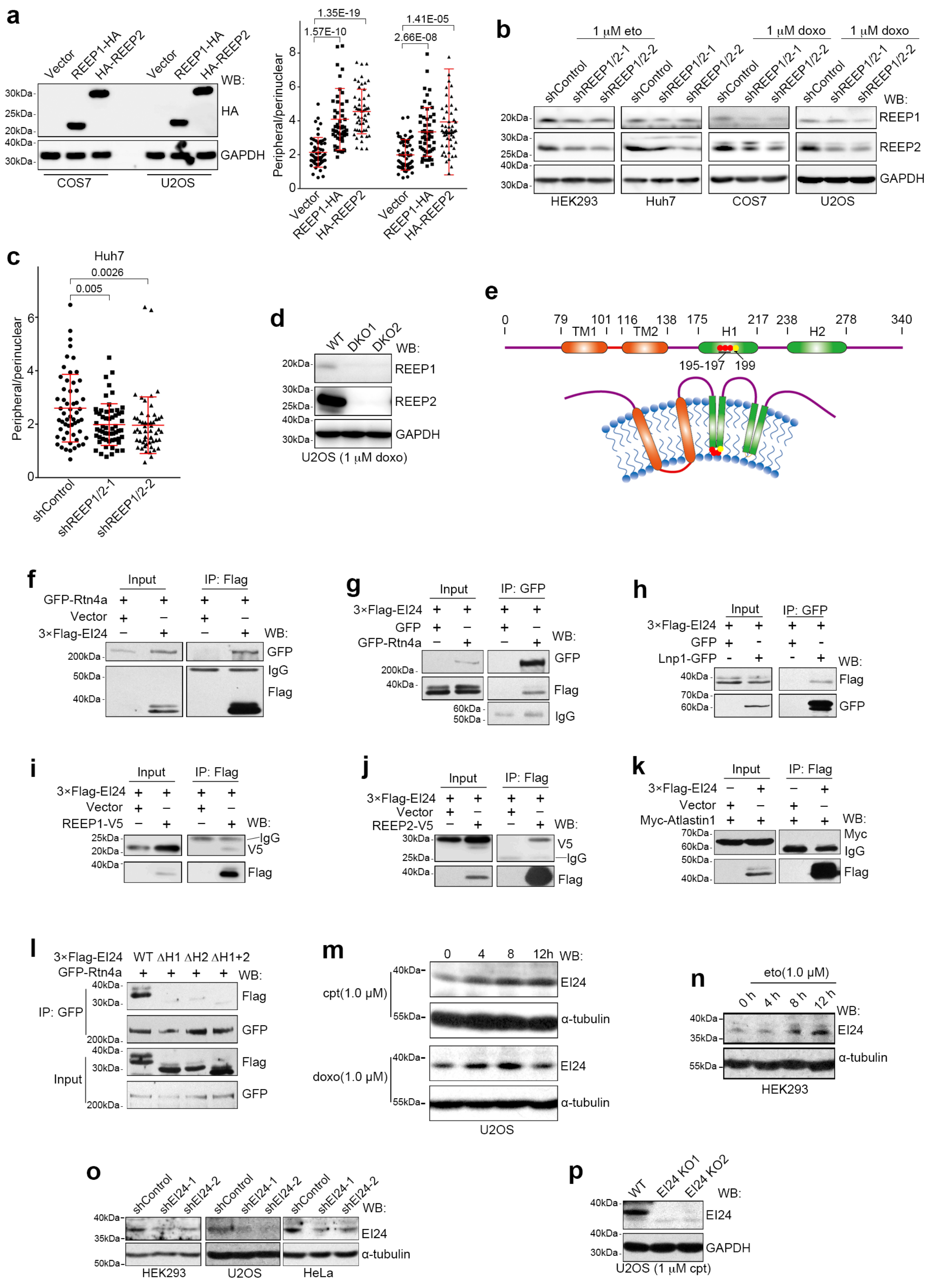
a
Vector
REEP1-HA
HA-REEP2
WB:
HA
GAPDH
COS7
U2OS
30kDa
25kDa
20kDa
40kDa
1.35E-19
1.57E-10
1.41E-05
2.66E-08
Peripheral/perinuclear
b
1 μM eto
1 μM doxo
shControl
shREEP1/2-1
shREEP1/2-2
REEP1
REEP2
GAPDH
HEK293
Huh7
COS7
U2OS
c
Huh7
0.0026
0.005
d
WT
DKO1
DKO2
U2OS (1 μM doxo)
e
0
79
101
116
138
175
217
238
278
340
TM1
TM2
H1
H2
195-197
199
f
Input
IP: Flag
GFP-Rtn4a
Vector
3×Flag-EI24
GFP
IgG
Flag
200kDa
50kDa
g
IP: GFP
GFP-Rtn4a
h
Lnp1-GFP
60kDa
70kDa
i
REEP1-V5
V5
j
REEP2-V5
k
Myc-Atlastin1
Myc
l
WT ΔH1 ΔH2 ΔH1+2
m
0 4 8 12h
cpt(1.0 μM)
doxo(1.0 μM)
EI24
α-tubulin
55kDa
n
eto(1.0 μM)
0 h
4 h
8 h
12 h
35kDa
o
shEI24-1
shEI24-2
HeLa
p
EI24 KO1
EI24 KO2
U2OS (1 μM cpt)

Figure S3. Depletion of REEP1/2 or EI24, and Association of EI24 with other ER-shaping Proteins.

(a) Distribution ratios (right panel) of peripheral/perinuclear ER in COS7 and U2OS cells overexpressing control (vector), REEP1-HA or HA-REEP2. $n \geq 50$ cells. Corresponding western blot (WB) analysis is shown in the left panel.

(b) Lysates from HEK293, Huh7, COS7, and U2OS cells transfected with control (shControl) or REEP1/2 shRNAs (shREEP1/2) were subjected to western blot analysis. HEK293 cells were treated with 1 μM eto for 16 hours. COS7 and U2OS cells were treated with 1 μM doxo for 16 hours. shREEP1/2 indicates co-transfection of shREEP1 and shREEP2.

(c) Distribution ratio of peripheral/perinuclear ER in Huh7 cells transfected with control (shControl) or REEP1/2 shRNAs (shREEP1/2). shREEP1/2 indicates co-transfection of shREEP1 and shREEP2. $n \geq 50$ cells.

(d) Western blot analysis of lysates from wild-type (WT) and REEP1/2 double-knockout (DKO) U2OS cells.

(e) Schematic of EI24 domains. Cytosolic sequences of EI24 are marked as purple lines, while luminal EI24 is marked as a red line. Two pathological point mutation sites in the first hairpin region are indicated as red or yellow dots. TM: regular transmembrane domain; H: intramembrane hairpin region.

(f-k) HEK293T cells co-overexpressing 3×Flag-EI24 together with GFP-Rtn4a (f and g), Lnp1-GFP (h), REEP1-V5 (i), REEP2-V5 (j), or myc-Atlastin1 (k) were subjected to immunoprecipitation (IP) and western blot as shown.

(l) HEK293T cells transfected with GFP-Rtn4a and wild-type or truncated 3×Flag-EI24 constructs were subjected to an immunoprecipitation assay and western blot at 36-hours post-transfection.

(m, n) Western blot analysis of lysates from U2OS cells treated with 1 μM cpt or 1 μM doxo (m) or else HEK293 cells treated with 1 μM eto (n) for the indicated times. Tubulin served as a loading control.

(o) Western blot analysis of lysates from HEK293, U2OS or HeLa cells transfected with control (shControl) or EI24 shRNA (shEI24) for 60 hours. Tubulin served as a loading control.

(p) Western blot analysis of lysates from wild-type and EI24 knockout (KO) U2OS cells treated with 1 μM cpt for 16 hours.
