# Supplementary material for: DNA damage triggers tubular endoplasmic reticulum extension to promote apoptosis by facilitating ER-mitochondria signaling
Source: Cell Res. 2018 Jul 20;28(8):833–54. doi: 10.1038/s41422-018-0065-z (PMC6063967; doi:10.1038/s41422-018-0065-z)
Supplement: Supplementary file 7 — Supplementary information, Figure S4 [file 41422_2018_65_MOESM7_ESM.pdf]

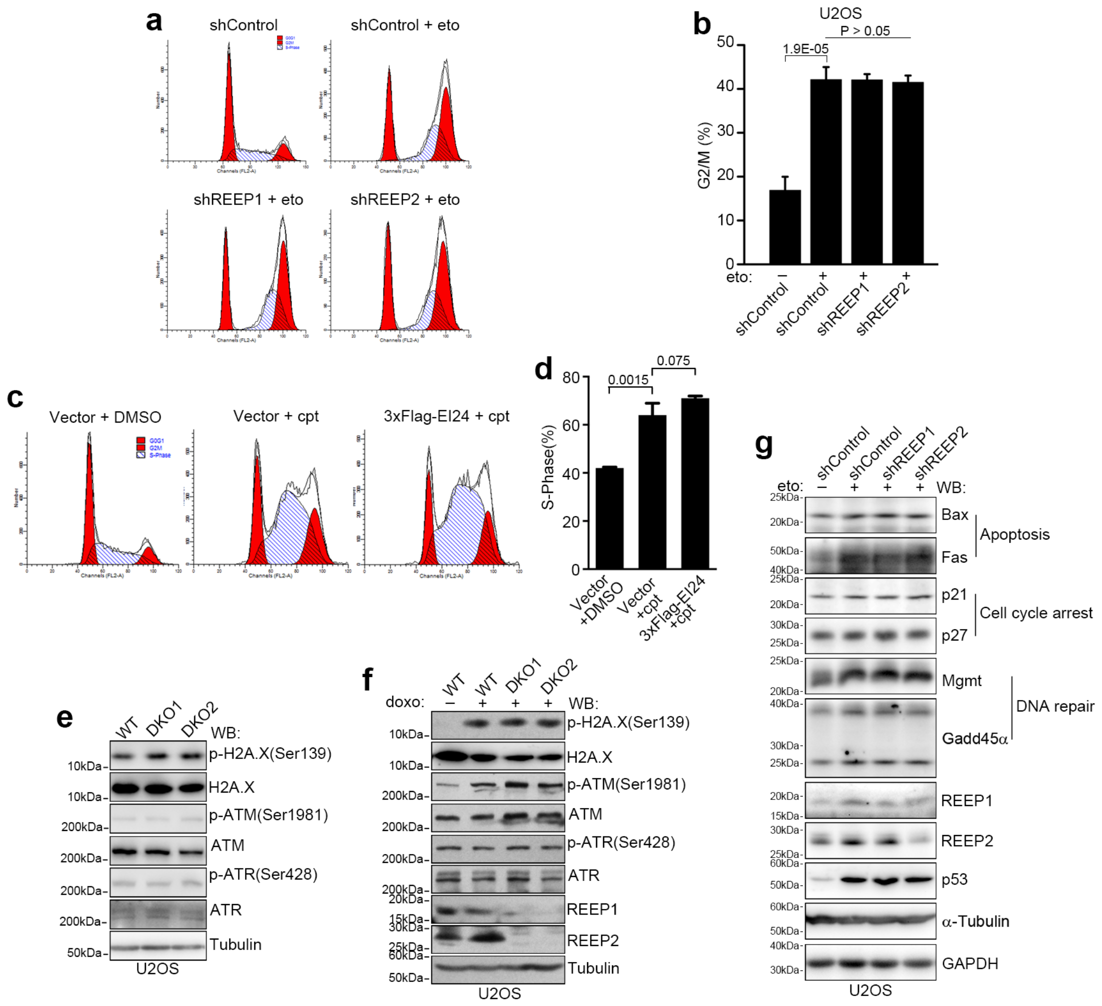

Figure S4. REEP1/2 and EI24 do not Affect Cell Cycle Arrest, DNA Repair or other p53 Downstream Pathways upon DNA Damage.

(a) Representative flow cytometry results of U2OS cells with REEP1/2 stable knockdown treated with DMSO or 5 μM eto for 16 hours.

(b) Statistical analysis of the percentage of G2/M cells from (a).

(c) Representative flow cytometry results for HEK293 cells transfected with the control vector or 3×Flag-EI24 and treated with DMSO or cpt for 12 hours.

(d) Statistical analysis of the percentage of S phase cells from (c).

(e) Western blot (WB) analysis of lysates from wild-type (WT) or REEP1/2 double knockout (DKO) U2OS cells (two different lines). Tubulin served as the loading control.

(f) Western blot analysis of lysates from U2OS cells with REEP1/2 double knockout treated with DMSO or 200 nM doxo for 16 hours.

(g) Western blot analysis of lysates from U2OS cells with REEP1/2 stable knockdown treated with DMSO or 5 μM eto for 16 hours. GAPDH and tubulin served as the loading controls.
