# Supplementary material for: DNA damage triggers tubular endoplasmic reticulum extension to promote apoptosis by facilitating ER-mitochondria signaling
Source: Cell Res. 2018 Jul 20;28(8):833–54. doi: 10.1038/s41422-018-0065-z (PMC6063967; doi:10.1038/s41422-018-0065-z)
Supplement: Supplementary file 8 — Supplementary information, Figure S5 [file 41422_2018_65_MOESM8_ESM.pdf]

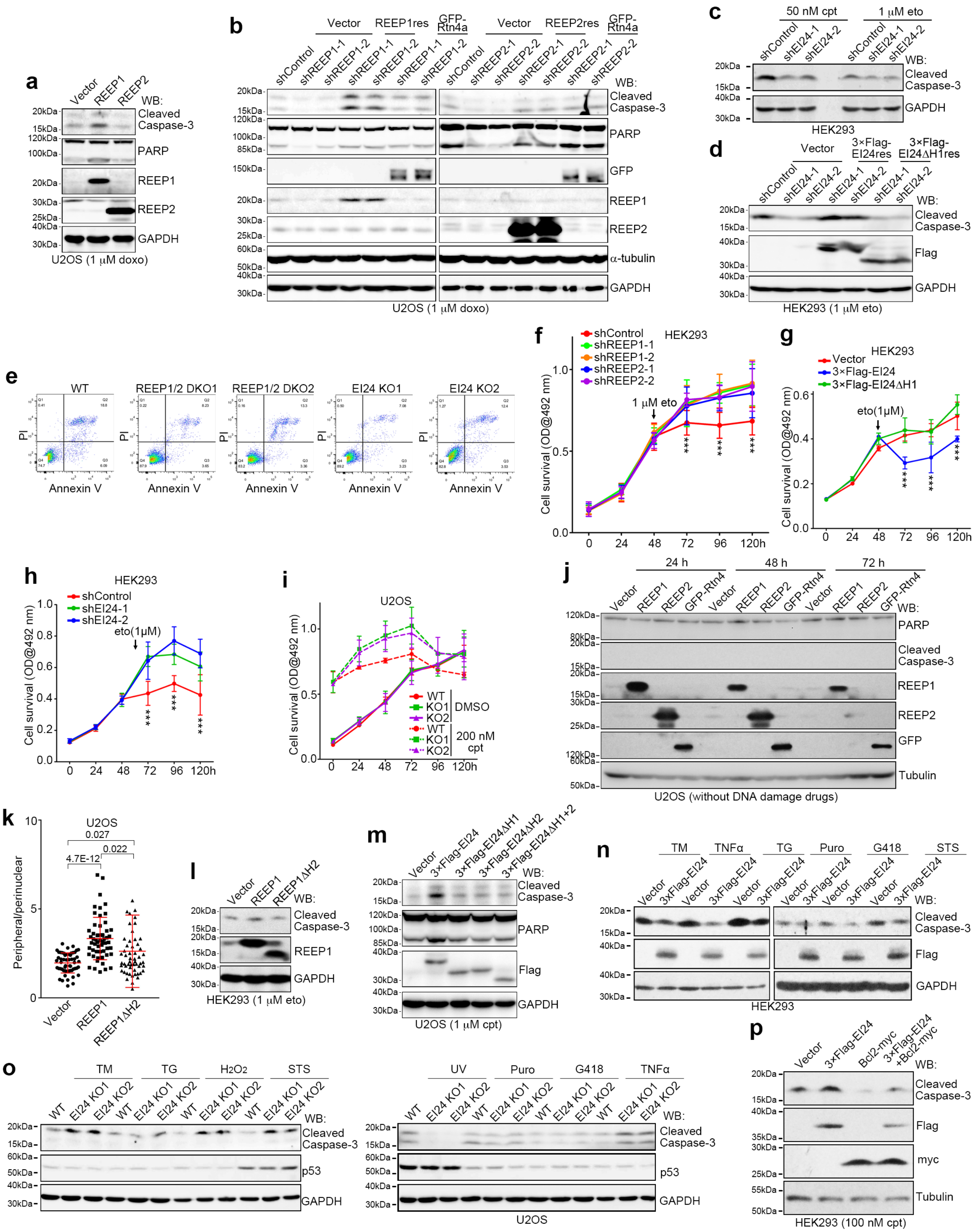
a
Vector
REEP1
REEP2
WB:
Cleaved Caspase-3
PARP
REEP1
REEP2
GAPDH
U2OS (1 μM doxo)
b
Vector
REEP1res
GFP-Rtn4a
REEP2res
shControl
shREEP1-1
shREEP1-2
shREEP2-1
shREEP2-2
WB:
Cleaved Caspase-3
PARP
GFP
REEP1
REEP2
α-tubulin
GAPDH
U2OS (1 μM doxo)
c
50 nM cpt
1 μM eto
shControl
shEI24-1
shEI24-2
WB:
Cleaved Caspase-3
GAPDH
HEK293
d
Vector
3×Flag-EI24res
3×Flag-EI24ΔH1res
WB:
Cleaved Caspase-3
Flag
GAPDH
HEK293 (1 μM eto)
e
WT
REEP1/2 DKO1
REEP1/2 DKO2
EI24 KO1
EI24 KO2
PI
Annexin V
f
shControl
shREEP1-1
shREEP1-2
shREEP2-1
shREEP2-2
HEK293
1 μM eto
Cell survival (OD@492 nm)
g
Vector
3×Flag-EI24
3×Flag-EI24ΔH1
HEK293
eto(1μM)
Cell survival (OD@492 nm)
h
HEK293
shControl
shEI24-1
shEI24-2
eto(1μM)
Cell survival (OD@492 nm)
i
U2OS
WT
KO1
KO2
DMSO
200 nM cpt
Cell survival (OD@492 nm)
j
24 h
48 h
72 h
Vector
REEP1
REEP2
GFP-Rtn4
WB:
PARP
Cleaved Caspase-3
REEP1
REEP2
GFP
Tubulin
U2OS (without DNA damage drugs)
k
U2OS
0.027
4.7E-12
0.022
Peripheral/perinuclear
Vector
REEP1
REEP1ΔH2
l
Vector
REEP1
REEP1ΔH2
WB:
Cleaved Caspase-3
REEP1
GAPDH
HEK293 (1 μM eto)
m
Vector
3×Flag-EI24
3×Flag-EI24ΔH1
3×Flag-EI24ΔH2
3×Flag-EI24ΔH1+2
WB:
Cleaved Caspase-3
PARP
Flag
GAPDH
U2OS (1 μM cpt)
n
TM
TNFα
TG
Puro
G418
STS
Vector
3xFlag-EI24
WB:
Cleaved Caspase-3
Flag
GAPDH
HEK293
o
TM
TG
H2O2
STS
UV
Puro
G418
TNFα
WT
EI24 KO1
EI24 KO2
WB:
Cleaved Caspase-3
p53
GAPDH
U2OS
p
Vector
3×Flag-EI24
Bcl2-myc
3×Flag-EI24 +Bcl2-myc
WB:
Cleaved Caspase-3
Flag
myc
Tubulin
HEK293 (100 nM cpt)

Figure S5. REEP1/2 and EI24 are Required for DNA Damage-induced Apoptosis.

(a) Western blot (WB) analysis of lysates from U2OS cells transfected with control vector, REEP1 or REEP2, and treated with 1 μM doxo for 24 hours.

(b) Western blot analysis of lysates from U2OS cells stably expressing control (shControl) or REEP1/2 shRNAs (shREEP1/2), transfected with the indicated plasmids, and treated with 1 μM doxo for 24 hours. GAPDH and tubulin served as the loading controls.

(c) Western blot analysis of lysates from HEK293 cells transfected with control or EI24 shRNA (shEI24) and treated with 50 nM cpt or 1 μM eto for 12 hours.

(d) Western blot analysis of HEK293 cells transfected with the indicated shRNA alone or together with either 3×Flag-EI24 or 3×Flag-EI24ΔH1 rescue plasmids and treated with 1 μM eto for 12 hours.

(e) Representative images of flow cytometry analysis for wild-type (WT), REEP1/2 double knockout (DKO), or EI24 knockout (KO) cells positive for propidium iodide (PI) or Annexin V. Cells were treated with 1 μM cpt for 14 hours.

(f) MTT assay of HEK293 cells transfected with REEP1/2 shRNAs and treated with 1 μM eto at 48 hours after transfection. n = 8.

(g) MTT assays of HEK293 cells transfected with the vector, 3×Flag-EI24 or 3×Flag-EI24ΔH1 and treated with eto 48 hours after transfection (n = 8).

(h) MTT assays of HEK293 cells transfected with control or EI24 shRNA (shEI24) and treated with eto 48 hours after transfection (n = 8).

(i) MTT assays of wild-type and EI24-knockout (KO) U2OS cells treated with DMSO or cpt (n = 8).

(j) Western blot analysis of lysates from U2OS cells transfected with control (vector), REEP1/2 or GFP-Rtn4 for 24, 48 or 72 hours.

(k) Distribution ratios of peripheral/perinuclear ER in U2OS cells overexpressing control (vector), REEP1 or REEP1ΔH2.

(l) Western blot analysis of lysates from HEK293 cells transfected with vector, REEP1 or REEP1ΔH2 and treated with 1 μM eto for 12 hours.

(m) Western blot analysis of lysates from U2OS cells transfected with the vector, 3×Flag-EI24, 3×Flag-EI24ΔH1, 3×Flag-EI24ΔH2 or 3×Flag-EI24ΔH1+2 and treated with 1 μM cpt for 24 hours.

(n) Western blot analysis of lysates from HEK293 cells transfected with control vector or 3×Flag-EI24, and treated with 1 μM TM, 100 ng/mL TNFα, 100 nM TG, 500 ng/mL puromycin (puro), 600 ug/mL G418, or 20 nM STS for 12 hours.

(o) Western blot analysis of lysates from wild-type or EI24 knockout U2OS cells treated with 1 μM TM, 100 nM TG, 1.5 mM H_2O_2, 20 nM STS, 500 ng/mL puromycin, 600 ug/mL G418, or 100 ng/mL TNFα for 24 hours, or with 40 J/m^2 UV radiation.

(p) Western blot analysis of HEK293 cells transfected with 3×Flag-EI24 and Bcl2-myc as indicated and treated with 100 nM cpt for 12 hours.
