# Supplementary material for: DNA damage triggers tubular endoplasmic reticulum extension to promote apoptosis by facilitating ER-mitochondria signaling
Source: Cell Res. 2018 Jul 20;28(8):833–54. doi: 10.1038/s41422-018-0065-z (PMC6063967; doi:10.1038/s41422-018-0065-z)
Supplement: Supplementary file 9 — Supplementary information, Figure S6 [file 41422_2018_65_MOESM9_ESM.pdf]

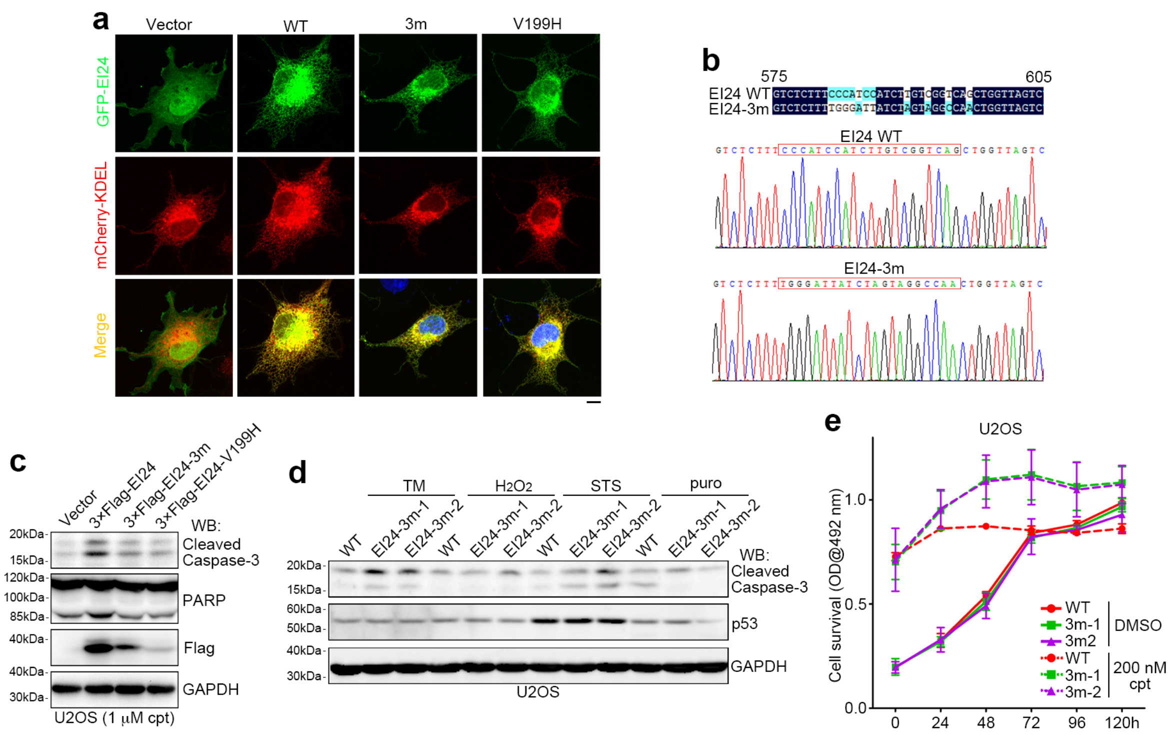

Figure S6. Effect of EI24 Pathological Mutation on ER Morphology and Apoptosis.

(a) Representative fluorescence images of COS7 cells transfected with mCherry-ER (red) and GFP-tagged wild-type or mutant EI24 constructs (green). Scale bar, 10 μm. DNA was stained with DAPI (blue).

(b) Sequence alignment of the partial EI24 coding sequences from EI24 wild-type and EI24-3m U2OS cells, and Sanger sequencing results showing the mutated EI24 region.

(c) Western blot (WB) analysis of U2OS cells overexpressing either EI24 or EI24 point mutants that were treated with 1 μM cpt for 24 hours.

(d) Western blot analysis of wild-type and EI24-3m U2OS cells treated with 2 μM TM, 1.5 mM H_2O_2, 20 nM STS or 500 ng/mL puromycin for 24 hours.

(e) MTT assays of wild-type and EI24-3m U2OS cells treated with DMSO or 200 nM cpt (n = 8).
