# Supplementary material for: DNA damage triggers tubular endoplasmic reticulum extension to promote apoptosis by facilitating ER-mitochondria signaling
Source: Cell Res. 2018 Jul 20;28(8):833–54. doi: 10.1038/s41422-018-0065-z (PMC6063967; doi:10.1038/s41422-018-0065-z)
Supplement: Supplementary file 10 — Supplementary information, Figure S7 [file 41422_2018_65_MOESM10_ESM.pdf]

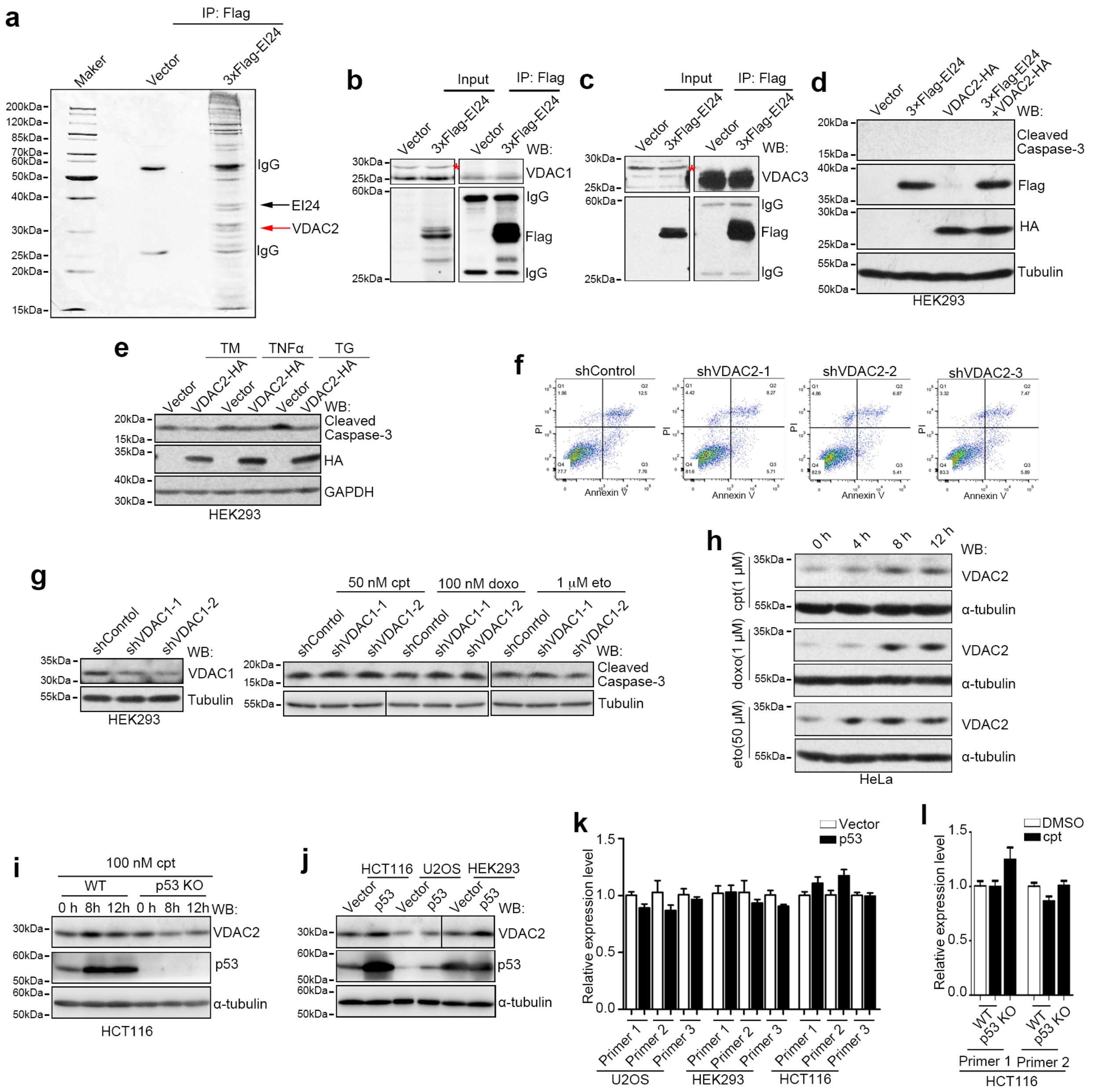

Figure S7. EI24 Specifically Interacts with VDAC2 and VDAC2 Protein Level is Increased Upon DNA Damage in a p53-dependent Manner.

(a) HEK293T cells transfected with the vector or 3×Flag-EI24 were immunoprecipitated (IP) with anti-Flag antibodies 36 hours after transfection, and the precipitated samples were analyzed by silver staining and mass spectrometry analysis. The black arrow indicates 3×Flag-EI24 enrichment, and the red arrow indicates VDAC2.

(b, c) HEK293T cells transfected with either the vector or 3×Flag-EI24 were immunoprecipitated with anti-Flag antibodies and subjected to western blot analysis with anti-Flag and either anti-VDAC1 (b) or anti-VDAC3 (c) antibodies.

(d) Western blot analysis of lysates from HEK293 cells transfected with 3×Flag-EI24 and VDAC2-HA, as

indicated.

(e) Western blot analysis of lysates from HEK293 cells transfected with either the control vector or VDAC2-HA and treated with 1 μM TM, 50 ng/mL TNFα or 100 nM TG for 12 hours.

(f) Representative flow cytometry analyses for VDAC2 shRNA-transfected cells positive for propidium iodide (PI) or Annexin V. Cells were treated with 100 nM cpt 12 hours.

(g) Western blot analysis of lysates from HEK293 cells transfected with control or VDAC1 shRNA and treated with 50 nM cpt, 100 nM doxo or 1 μM eto for 12 hours.

(h) Western blot analysis of lysates from HeLa cells treated with cpt, doxo or eto for the indicated times.

(i) Western blot (WB) analysis of lysates from wild-type (WT) or p53-knockout (KO) HCT116 cells treated with 100 nM cpt for the indicated times. Tubulin served as a loading control.

(j) Western blot analysis of lysates from HCT116, U2OS and HEK293 cells transfected with the vector or p53.

(k) U2OS, HEK293 and HCT116 cells were transfected with the control vector or p53 for 24 hours, and relative VDAC2 mRNA levels were analyzed by quantitative real-time PCR (n = 8).

(l) Wild-type (WT) or p53-knockout HCT116 cells were treated with DMSO or cpt for 12 hours, and the relative VDAC2 mRNA levels were analyzed by quantitative real-time PCR (n = 8).
