# Supplementary material for: DNA damage triggers tubular endoplasmic reticulum extension to promote apoptosis by facilitating ER-mitochondria signaling
Source: Cell Res. 2018 Jul 20;28(8):833–54. doi: 10.1038/s41422-018-0065-z (PMC6063967; doi:10.1038/s41422-018-0065-z)
Supplement: Supplementary file 11 — Supplementary information, Figure S8 [file 41422_2018_65_MOESM11_ESM.pdf]

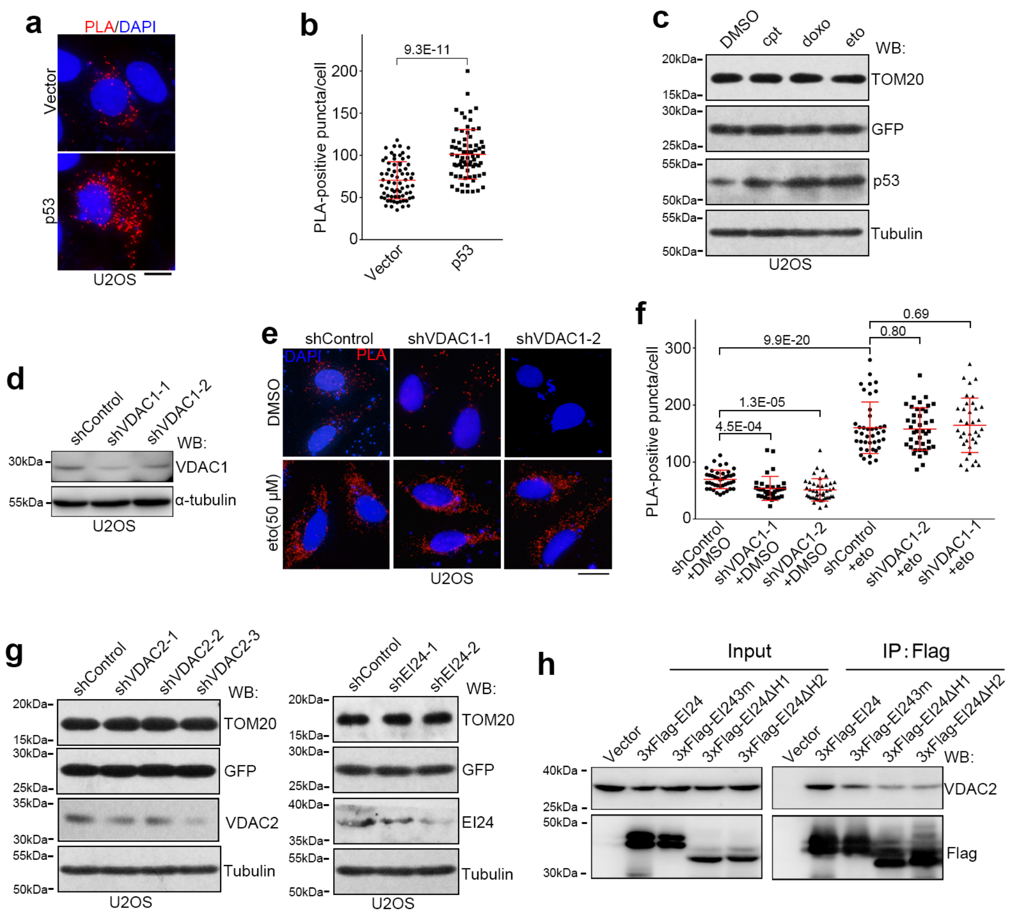

Figure S8. Control Data for DNA Damage Promotes ER-mitochondria Contacts by EI24 and VDAC2

(a) Representative images of the PLA (red) results for U2OS cells transfected with vector or p53. DNA was stained with DAPI (blue). Scale bar, 10 μm.

(b) Statistical analysis of the number of PLA-positive puncta per cell in (a).

(c) Western blot (WB) analysis of lysates from U2OS cells treated with DMSO, 1 μM cpt, 1 μM doxo or 50 μM eto for 12 hours.

(d) Western blot analysis of lysates from U2OS cells transfected with control (shControl) or VDAC1 shRNA (shVDAC1) for 60 hours.

(e) U2OS cells stably expressing CFP-ER were transfected with control (shControl) or VDAC1 shRNA (shVDAC1), treated with DMSO or eto, probed with anti-TOM20 and anti-GFP antibodies, and subjected to PLA. Scale bar, 10 μm.

(f) Quantification of the number of PLA-positive puncta/cell in (e).

(g) Western blot analysis of lysates from U2OS cells transfected with the indicated shRNAs.

(h) HEK293T cells transfected with the control vector, 3×Flag-EI24, 3×Flag-EI243m, 3×Flag-EI24ΔH1 or 3×Flag-EI24ΔH2 were subjected to immunoprecipitation (IP) assays and western blot analysis with the indicated antibodies.
