# Supplementary material for: DNA damage triggers tubular endoplasmic reticulum extension to promote apoptosis by facilitating ER-mitochondria signaling
Source: Cell Res. 2018 Jul 20;28(8):833–54. doi: 10.1038/s41422-018-0065-z (PMC6063967; doi:10.1038/s41422-018-0065-z)
Supplement: Supplementary file 12 — Supplementary information, Figure S9 [file 41422_2018_65_MOESM12_ESM.pdf]

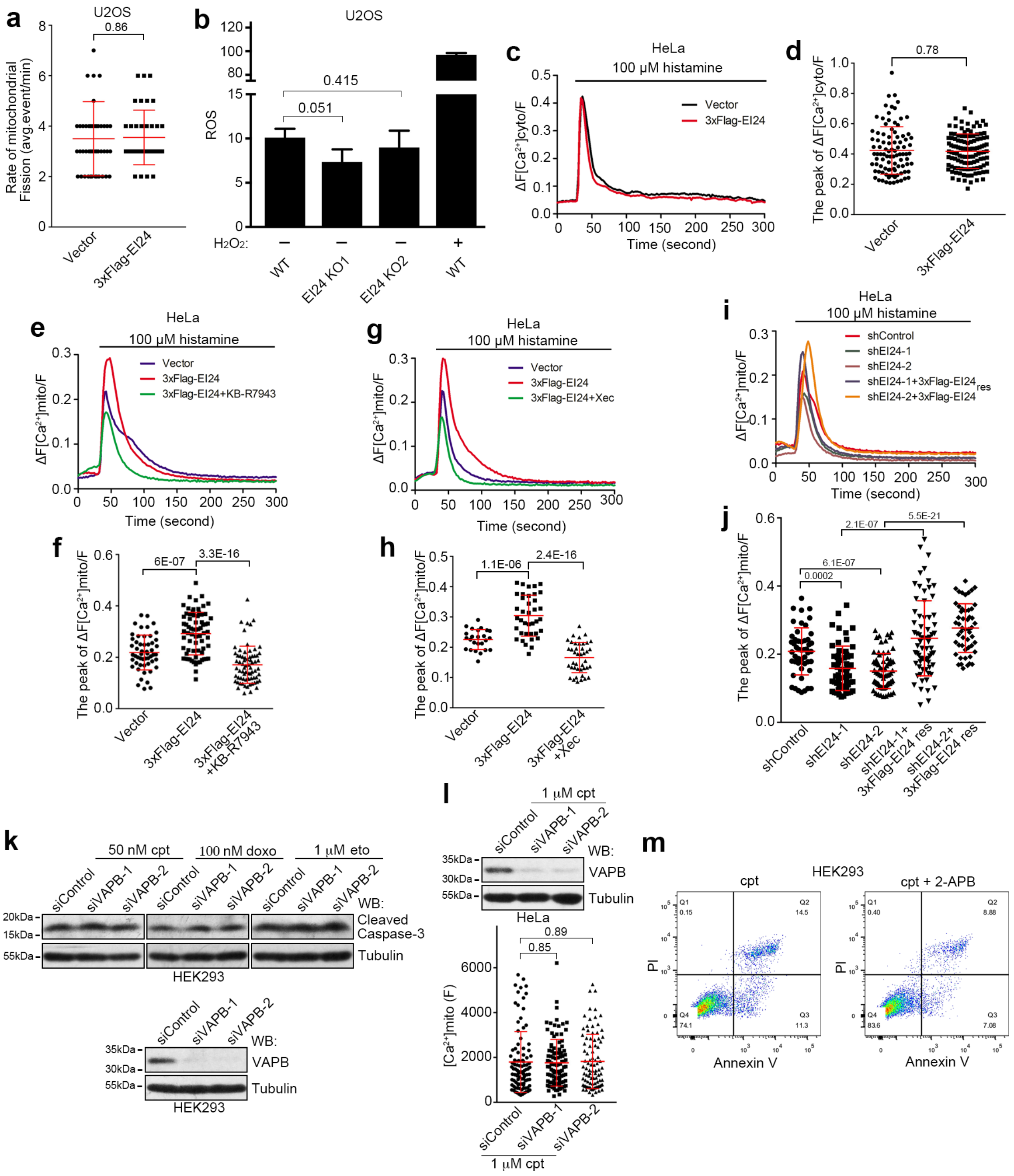

Figure S9. EI24 Promotes ER-mitochondria Ca^{2+} Flux.

(a) Mitochondrial fission rate of U2OS cells transfected with the vector or 3×Flag-EI24 (n > 50 cells). The rate of mitochondrial fission was calculated by determining the average number of fission events/min for each time point.

(b) ROS levels of wild-type (WT) and EI24 knockout (KO) cells with or without H_2O_2 treatment (50 µM, 30 min).

(c) GCaMP6s fluorescence assay to measure cytosolic Ca^{2+} after histamine stimulation in a vector- or EI24-

overexpressing (3×Flag-EI24) HeLa cells stably expressing cyto-GCaMP6s.

(d) Quantification of the cyto-GCaMP6s peak intensity from (c).

(e) HeLa cells stably expressing mito-R-GECO1.2 (mt-R-GECO1.2, red) were transfected with the vector or 3×Flag-EI24 and either treated with the MCU inhibitor KB-R7943 (10 μM) or left untreated. The traces show the mean ratio (ΔF/F) of the increased fluorescence intensity (ΔF) to the background fluorescence intensity (F) of the cells.

(f) Quantification of the mito-R-GECO1.2 peak intensity from (e).

(g) Measurement of mito-R-GECO1.2 fluorescence intensity, which indicates mitochondrial Ca^{2+}, following histamine stimulation in the control (Vector) and EI24 overexpressing (3×Flag-EI24) HeLa cells that stably expressed mito-R-GECO1.2 and were either treated with the IP3Rs inhibitor Xec (1 μM) or left untreated.

(h) Quantification of the mito-R-GECO1.2 peak intensity from (g).

(i) Measurement of the mito-R-GECO1.2 fluorescence intensity after histamine stimulation in control (shControl), EI24 knockdown (shEI24), and EI24 knockdown-rescued (shEI24 + 3×Flag-EI24res) HeLa cells stably expressing mito-R-GECO1.2.

(j) Quantification of the mito-R-GECO1.2 peak intensity from (i).

(k) Western blot (WB) analysis of lysates from HEK293 cells transfected with control or VAPB siRNA and treated with 50 nM cpt, 100 nM doxo or 1 μM eto for 12 hours.

(l) Fluorescence intensity of HeLa cells stably expressing mito-R-GECO1.2, transfected with control or VAPB siRNAs, and treated with 1 μM cpt for 10 hours. The corresponding western blot is shown in the upper panel.

(m) Representative images of flow cytometry data for cells positive for propidium iodide (PI) or Annexin V. Cells were treated with 100 nM cpt and 10 μM KB-R7943 for 16 hours.
