# Supplementary material for: DNA damage triggers tubular endoplasmic reticulum extension to promote apoptosis by facilitating ER-mitochondria signaling
Source: Cell Res. 2018 Jul 20;28(8):833–54. doi: 10.1038/s41422-018-0065-z (PMC6063967; doi:10.1038/s41422-018-0065-z)
Supplement: Supplementary file 13 — Supplementary information, Figure S10 [file 41422_2018_65_MOESM13_ESM.pdf]

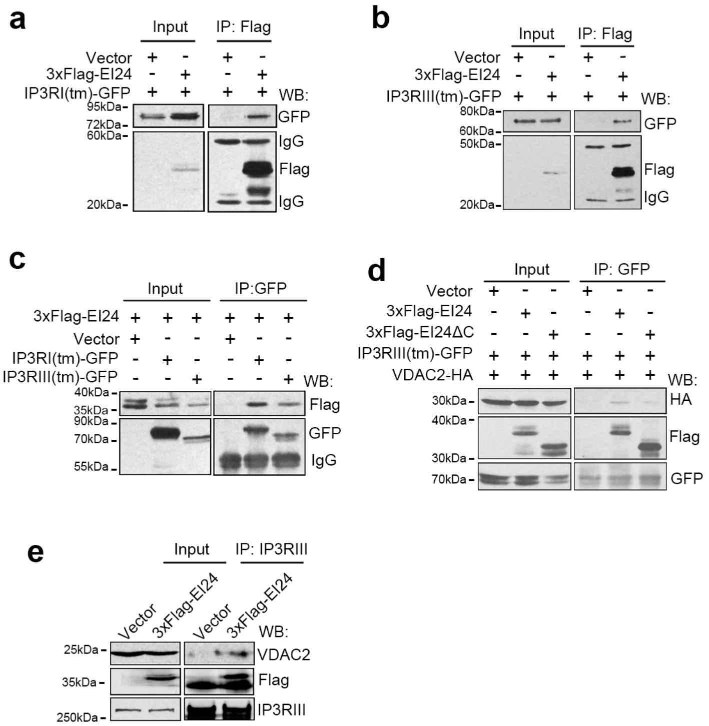

Figure S10. EI24 Promotes the Association between IP3R and VDAC2.

(a-c) HEK293T cells transfected with 3×Flag-EI24 and IP3RI (tm)-GFP or IP3RIII (tm)-GFP (the transmembrane domain of IP3RI or IP3RIII tagged with GFP) were subjected to immunoprecipitation (IP) with anti-Flag or anti-GFP antibodies and western blot (WB) analysis.

(d) HEK293T cells transfected with IP3RIII (tm)-GFP, VDAC2-HA, and 3×Flag-EI24 or 3×Flag-EI24ΔC were subjected to immunoprecipitation with anti-GFP antibodies and western blot analysis.

(e) HEK293T cells transfected with 3×Flag-EI24 were subjected to immunoprecipitation with an anti-IP3RIII antibody and western blot analysis with anti-VDAC2 and anti-Flag antibodies.
