# Supplementary material for: DNA damage triggers tubular endoplasmic reticulum extension to promote apoptosis by facilitating ER-mitochondria signaling
Source: Cell Res. 2018 Jul 20;28(8):833–54. doi: 10.1038/s41422-018-0065-z (PMC6063967; doi:10.1038/s41422-018-0065-z)
Supplement: Supplementary file 14 — Supplementary Information, Table S1 [file 41422_2018_65_MOESM14_ESM.pdf]

Table S1. Primers Used for qRT-PCR

F-RT-Atlastin1	CCAAATGACTTGCAGACCAAAC
R-RT-Atlastin1	CCCTCGGAATAGCTTCACAGA
F-RT-Atlastin2	CGTACCCCAGCCACACTGT
R-RT-Atlastin2	TGAAGCCAGTCAGTCCTGAGATT
F-RT-Atlastin3	CCGATGTCACCTGCTTTCTCTT
R-RT-Atlastin3	CCATCAAAGTCAGGGCTTGTG
F-RT-Climp63	TGGAGGCGGACTTGAAAATG
R-RT-Climp63	ATTTTGACCGAGTATGCAACCA
F-RT-Kinectin	GGAGCAGAATGAGGCTTTGAA
R-RT-Kinectin	CGGAGGTCTGGGCTGCTA
F-RT-Lnp1	GCCCTATCATCAAATGTGTTACCA
R-RT-Lnp1	CCCATTCCAGGCACTGAAGT
F-RT-p180	CCAGAGGCACCAAAGCAAGA
R-RT-p180	GGGCCCAGGCTCACCTT
F-RT-Protrudin	GATGAACCCAAAGCAGGAAGAG
R-RT-Protrudin	ATCCTTCCCCCCAACATCTG
F-RT-Rab3GAP1	GGGAAACTTACACTGCTGCATAATG
R-RT-Rab3GAP1	AGGTGCTGGTTCCTGGGTTA
F-RT-Rab3GAP2	CAAGCCAAAAGAGGAGTTCCA
R-RT-Rab3GAP2	AAGTAAAACCCACCACAATGCA
F-RT-Rab10	AGGCCAGGAGCGATTTCAC
R-RT-Rab10	ACCCATTGCGCCTCTGTAGT
F-RT-Rab18	TCCTCATCATCGGCGAGAGT
R-RT-Rab18	AACGTATCATCTGTGAACCTCAAGAG
F-RT-REEP1	GGGACAGGGTGCCTTATCG
R-RT-REEP1	TGGTGAGGTCCTGCATGCT
F-RT-REEP2	TGGCTGCTGTCCCCTTACA

R-RT-REEP2	GTGCACGAACTTGCGGTAGA
F-RT-REEP3	GTTGCTTGGTTTCCCCTGTACT
R-RT-REEP3	GGAGAAAGCAGCCATATGACAAA
F-RT-REEP4	CCTGCTTTACCGCAAGTTTGT
R-RT-REEP4	CGCGTCGATCTCCTTCTCAT
F-RT-REEP5	CACGAGTCCCAGATGGACAGT
R-RT-REEP5	CTGCAGTCTCTTTGGCCTTGT
F-RT-REEP6	AAGTGCGCCTTCCTGTTGTT
R-RT-REEP6	CATGAGAGCCCCGTTCCA
F-RT-Rtn1	GGCCTACTTGGAGCTTGAGATC
R-RT-Rtn1	GCAGGCAGTCCGTGTACTTCT
F-RT-Rtn2	GGTCTTCGCCCACTGCAA
R-RT-Rtn2	CCTTCTGTGGAATCAGGAGTTGA
F-RT-Rtn3	GACTCCTTGAAGCTGGCTGTCT
R-RT-Rtn3	TCCGTTAAAAACAGCACCAACA
F-RT-Rtn4	ACACTACTGATTTTGGCTCTCATTTC
R-RT-Rtn4	TGTGCCTGATGCCGTTCAT
F-RT-Spastin	CCACCTGGGAATGGGAAGA
R-RT-Spastin	TTGCATTCGATTCTGCAGCTA
F-RT-STIM1	TGGGATCTCAGAGGGATTTGA
R-RT-STIM1	GCTGGCGGTCACTCATGTG
F-RT-EI24	GAATGCCATTTGGTTTCAGGAT
R-RT-EI24	TGAGGCTTCCTCCCTGATACC
F-RT-VDAC2-1	CCTTGGTTGTGATGTTGACTTTGA
R-RT-VDAC2-1	CTGATGTCCAAGCAAGGTTTACTG
F-RT-VDAC2-2	TTGGATCCCACTGCTTCCATTTCTGCAAA
R-RT-VDAC2-2	ATTAATGCTCTTCCCATCTACCAGAGCAGA
F-RT-VDAC2-3	CTGGGAACAGAAATCGCAA

R-RT-VDAC2-3	TGAACCATGGATTGCAGGT
F-RT-GAPDH	GGCATCCTGGGCTACACTGA
R-RT-GAPDH	GGAGTGGGTGTCGCTGTTG
